# Web-Based Interventions for Pregnant Women With Gestational Diabetes Mellitus: Systematic Review and Meta-analysis

**DOI:** 10.2196/36922

**Published:** 2023-01-19

**Authors:** Pingping Guo, Dandan Chen, Ping Xu, Xiaojuan Wang, Wei Zhang, Minna Mao, Qiong Zheng, Ying Jin, Suwen Feng

**Affiliations:** 1 Women’s Hospital, Zhejiang University School of Medicine Hangzhou, Zhejiang Province China; 2 Faculty of Nursing, Zhejiang University School of Medicine Hangzhou China

**Keywords:** gestational diabetes mellitus, internet, intervention, meta-analysis, pregnant women, systematic review, mobile phone

## Abstract

**Background:**

Effective management of gestational diabetes mellitus (GDM) is essential because it is one of the most prevalent diseases during pregnancy, and the consequent condition maternal hyperglycemia is closely related to considerable short- and long-term maternal and neonatal complications. Web-based interventions (WBIs), defined as therapeutic interventions offered via the web, have been implemented to assist in managing GDM owing to their advantages of high accessibility and efficiency, but findings across relevant studies are inconsistent.

**Objective:**

This systematic review and meta-analysis aimed to evaluate the effectiveness of WBIs on glycemic control among pregnant women with GDM; examine whether specific types of intervention interactivity, format, and technology have beneficial effects on maternal glycemic control; and comprehensively assess the efficacy of WBIs in maternal behavioral outcomes, cognitive and attitudinal outcomes, mental health, maternal and neonatal clinical outcomes, and medical service use and costs among pregnant women with GDM.

**Methods:**

PubMed, Embase, Cochrane Library, Web of Science, CINAHL, and PsycINFO were searched from their respective inception to November 19, 2022, to identify relevant randomized controlled trials and controlled clinical trials. The methodological quality of the included studies was assessed using the Effective Public Health Practice Project tool. Where possible, the data for all outcomes were meta-analyzed using the Stata software (version 12.0; StataCorp). Overall, 3 subgroup analyses and post hoc sensitivity analyses of maternal glycemic control parameters were performed.

**Results:**

Overall, 25 publications arising from 21 randomized controlled trials and controlled clinical trials were included. The overall meta-analyses on glycemic control parameters demonstrated that WBIs could significantly improve fasting blood glucose (standardized mean difference=−1.764, 95% CI −2.972 to −0.557; *P*=.004) and 2-hour postprandial blood glucose (standardized mean difference=−1.433, 95% CI −2.561 to −0.304; *P*=.01) compared with the control group, whereas no significant effect was found on glycated hemoglobin and 1-hour postprandial blood glucose. The results of the subgroup analyses indicated that mobile app–delivered interventions with a personalized format and interactive function showed more beneficial effects on maternal glycemic control. Moreover, WBIs could significantly enhance compliance with the self-monitoring of blood glucose; increase the rate of normal vaginal delivery; and decrease the chance of emergency cesarean, admission to the neonatal intensive care unit, and composite neonatal complications. GDM knowledge, risk perception of the disease, self-efficacy, satisfaction with care, and medical service use of the participants in the WBI group were also improved compared with the control group. However, the effectiveness of WBIs on other secondary outcomes was either nonsignificant or uncertain.

**Conclusions:**

WBIs are a promising approach to GDM management. Personalized, interactive, and mobile app–delivered interventions seem more worthy of being recommended for future clinical practice. Further high-quality studies are required to verify these findings before making broad recommendations.

**Trial Registration:**

PROSPERO CRD42022296625; https://www.crd.york.ac.uk/prospero/display_record.php?RecordID=296625

## Introduction

### Background

Gestational diabetes mellitus (GDM), one of the most prevalent complications during pregnancy, is defined as glucose intolerance and consequent hyperglycemia with onset or first recognition in the second or third trimester of pregnancy. It is mainly caused by the lack of sufficient insulin secretion from beta cells to match the increased insulin tolerance that develops under the influence of pregnancy hormones as pregnancy progresses [1]. Over the last 2 decades, the prevalence of GDM has increased by >30% in several countries [2]. According to global statistics, the incidence of GDM ranged from 7.5% to 27% up to 2019 [3]. Worse still, it is anticipated to grow even further as a consequence of the increasing rate of obesity, advanced maternal age, sedentary lifestyle, and the introduction of a more stringent clinical guideline for GDM diagnosis [1,4]. GDM has become one of the key public health issues in both high-income and low-income countries [5]. Evidence from intensive studies has established a close association between GDM and considerable maternal and neonatal complications, including preeclampsia, macrosomia, cesarean, shoulder dystocia, and neonatal hypoglycemia [6]. Importantly, the risk of these complications increases in lockstep with maternal glycemic levels [7]. Even worse is that although GDM occurs only during the peripartum period as a transient condition and gets resolved within a short period after delivery, the potential risk of consequent complications is not limited to pregnancy outcomes. Several studies have demonstrated that mothers with a history of GDM are more susceptible to its recurrence in subsequent pregnancies [8]. Moreover, GDM is an independent risk factor for many chronic illnesses that affect both women and their offspring in later life, including cardiovascular diseases and metabolic syndrome [9,10]. These short- and long-term negative consequences not only endanger the physical and mental health of mothers and children [11] but also incur a heavy financial burden on families and the society [12]. Therefore, effective measures should be taken to manage and treat GDM.

At present, a *step–up* approach is commonly applied to GDM management and has yielded great achievements; specifically, lifestyle interventions (mainly involving dietary modification and physical activity) are considered the first-line intervention [13], and insulin is added to the regimen to improve the treatment effect if the first-line intervention fails to maintain maternal glycemia at a safe level [14]. However, as most patients do not have sufficient knowledge about GDM, traditional GDM management entails frequent prenatal visits as well as close multidisciplinary follow-ups for education, counseling, reporting symptoms and blood glucose levels, and adjusting treatment regimens [15-17], which require intensive clinical input and can challenge the medical resources [11,15]. Meanwhile, many barriers (such as unequal health resource distribution, high medical costs, inconvenient traffic, time constraints, and a noisy clinical environment) have posed great challenges to the traditional mode of GDM management and have decreased patients’ satisfaction [11,17,18]. What also cannot be ignored is that complications may occur before doctors can take any action owing to the lag in information caused by the interval between 2 prenatal visits [19]. Hence, there is an urgent need to introduce innovative and sustainable modes of health care to help manage GDM effectively with minimal burdens and disruptions for patients and health care systems.

Web-based interventions (WBIs), which are defined as therapeutic programs with specific health objectives delivered using web-connected devices, seem to be an ideal mode of medical and public health practice in the era of information and communication technology revolution, as they contribute to closing the loop between patients and health care providers; realizing the vision of pervasive health care; overcoming the inequivalent distribution of medical resources; and increasing the accessibility, continuity, and efficiency of medical services [19,20]. In recent years, WBIs have drawn great attention from the medical and hygiene fields and have been widely integrated into health systems to assist in the management of various diseases [21-23]. Women of childbearing age are regarded as an ideal target population for the implementation of web-based technologies that improve their health, as they generally own at least 1 web-based device and have an excellent grasp of these technologies [16,24,25]. Many studies have investigated the effectiveness of WBIs in pregnant women with GDM and indicated improvements in glycemic control [19,26], antenatal anxiety [27], compliance with the self-monitoring of blood glucose (SMBG) [19], incidence of premature delivery [28], medical service costs [15], and satisfaction with care [16]. However, research findings are conflicting, because some studies have discovered null relationships between WBIs and the aforementioned outcomes among this population [29-31]. Therefore, a systematic evaluation of the efficacy of WBIs in pregnant women with GDM is essential.

### Prior Work

To date, 2 systematic reviews [32,33] have been conducted to investigate the effectiveness of WBIs in pregnant women with GDM. One of the reviews [32] included perinatal diabetes (GDM, type 1 diabetes, and type 2 diabetes), and the results of the subgroup analysis of GDM (n=5 studies) revealed no significant between-group differences in glycated hemoglobin (HbA_1c_), cesarean rate, neonatal birth weight, or hypoglycemia. By contrast, the other review (n=6 studies) [33], which was conducted recently, demonstrated that fasting blood glucose (FBG), 2-hour postprandial blood glucose (2hBG), and cesarean rate significantly improved among pregnant women with GDM after the use of disease-specific mobile app interventions compared with the control. There are 5 additional systematic reviews [23,34-37] regarding the efficacy of telemedicine in GDM, which included both health interventions delivered by the internet and early technologies such as phone calls, short messages, emails, and digital video disks; however, 2 of them [36,37] were published 5 years ago and involved a limited number of primary studies (n≤6). A recent review by Xie et al [34] showed that telemedicine could significantly ameliorate HbA_1c_, FBG, 2hBG, and some maternal and neonatal clinical outcomes in pregnant women with GDM; however, approximately 60% of the trials analyzed in this review were from China, which might cause regional bias and influence the external validity of the findings. A recent review by Eberle et al [35] assessed the effects of telemedicine on only HbA_1c_ and FBG among pregnant women with GDM in the context of the COVID-19 pandemic and revealed favorable impacts. Nonetheless, Li et al [23] focused on the effectiveness of telemedicine-based lifestyle interventions for GDM but reported a significant reduction in only 2hBG compared with the control group. Collectively, the existing systematic reviews on relevant topics yielded mixed results regarding the effect of WBIs on maternal glycemic control and clinical outcomes, whereas the effects on other outcomes (such as maternal behavioral outcomes and medical service use and costs) were scarcely discussed. In addition, most of them [23,34-37] conflated web-based technologies with early mobile technologies, which are labor intensive and have become less popular under the rapidly evolving landscape of technology. More importantly, many primary trials [15,29,30,38-45] on this topic with conflicting results emerged after these reviews, which might provide new evidence. Consequently, a new systematic review is necessary to comprehensively investigate the effectiveness of WBIs in pregnant women with GDM based on all the existing evidence from randomized controlled trials (RCTs) and controlled clinical trials (CCTs) that meet the high standards of evidence-based research, thereby providing health practitioners with scientific evidence and all-round contextual information regarding this topic and aiding implementation decisions in clinical settings.

### Goal of This Study

Given that maternal hyperglycemia is the pathological basis for GDM complications and that effective glycemic control is the most key link in GDM management, the primary objective of this systematic review was to assess the effectiveness of WBIs on maternal glycemic control in pregnant women with GDM. Moreover, 3 subgroup analyses were performed to investigate the influence of the type of interactivity, format, and technology of WBIs on glycemic control in this population. The secondary objective was to examine whether WBIs had beneficial effects on other broad outcomes in pregnant women with GDM, including maternal behavioral outcomes, cognitive and attitudinal outcomes, mental health, maternal and neonatal clinical outcomes, and medical service use and costs.

## Methods

### Ethical Considerations

This review was exempt from institutional review board approval because no human participants were involved. The methods used for reporting the results of this study are in compliance with the PRISMA (Preferred Reporting Items for Systematic Reviews and Meta-Analyses) guidelines [46]. Multimedia Appendix 1 [46] presents the PRISMA checklist. The research protocol was registered in PROSPERO (registration number CRD42022296625) and published elsewhere [47].

### Information Sources and Literature Search

Two reviewers independently performed 2 waves of literature searches in 6 English-language electronic databases (PubMed, Embase, Cochrane Library, Web of Science, CINAHL, and PsycINFO). An initial search was conducted on January 26, 2022, and an updated search was conducted on November 19, 2022. Search strategies for all databases, including a combination of medical subject heading terms and entry terms to represent the definitions of WBIs, GDM, RCTs, and CCTs (refer to Multimedia Appendix 2 for details), were customized and developed in collaboration with 2 academic librarians at the first author’s university. In addition, we manually searched the reference lists of all the included studies and relevant systematic reviews to identify additional eligible studies.

### Eligibility Criteria

Studies were included if they met all the following criteria. First, the participants were pregnant women aged ≥18 years with GDM in current pregnancy, regardless of whether they had been diagnosed with GDM previously. Pregnant women with various types of diabetes were included, but the outcomes of GDM were reported separately. Second, the intervention was a digital one delivered via a web-based modality and conducted based on internet-connected devices such as a smartphone, computer, and laptop, which could include any web-based series of curriculum, instructions, lessons, modules, options, or plans. Third, the control group comprised participants in a waitlist, those receiving usual care, or those not receiving treatment. Fourth, the primary outcomes were glycemic control indicators, including the levels of HbA_1c_, FBG, 1-hour postprandial blood glucose (1hBG), and 2hBG, and the secondary outcomes were maternal behavioral outcomes (compliance with SMBG, healthy diet behaviors, and physical activity), maternal cognitive and attitudinal outcomes (knowledge of disease, risk perception of disease, self-efficacy, and satisfaction with care), maternal mental health (depression and anxiety), maternal and neonatal clinical outcomes (eg, premature delivery and macrosomia), and medical service use and costs. Studies that evaluated at least one of the above outcomes were eligible. Finally, the study should have been an RCT or a CCT published in a peer-reviewed English journal.

The exclusion criteria were as follows. First, the participants were pregnant women with type 1 diabetes, type 2 diabetes, impaired glucose tolerance, severe diseases, severe symptoms of psychological disorders (eg, bipolar disorder and psychotic disorder), or fetal abnormalities. Second, there was a lack of real WBIs for participants or minimal WBIs, such as interventions using a digital video disk; a radio; a short message; a television; a video; telephone calls; a video phone; purely videoconferencing; or a weblink only to a digital video, audio, picture, or text. WBIs were implemented only as follow-up interventions or for assessment purposes to observe the maintenance effects of previously administered health interventions. Studies combining WBIs with traditional face-to-face interventions were excluded because it was difficult to distinguish whether outcome changes were attributable to web-based components. Studies investigating the efficacy of continuous glucose monitoring systems via portable sensors were beyond the scope of this review. Third, the outcomes of interest were lacking or were measured at postpartum. Finally, single-group studies, reviews, case reports, cohort studies, letters, conference abstracts, and study protocols were excluded.

### Study Selection and Data Extraction

After removing duplicates using EndNote (version X8.2, Clarivate Plc), 2 reviewers independently screened the retrieved titles and abstracts and, ultimately, the full text for eligibility. Any disagreement was resolved through discussion with a third reviewer. Subsequently, 2 reviewers independently conducted data extraction using a predesigned Excel (Microsoft Corp) worksheet. Then, another reviewer checked the accuracy of the extracted data. Specifically, we extracted the following information from each included study: general information (first author, year of publication, country, and study design), participant characteristics (gestational weeks, diagnostic criteria of GDM, sample size, and mean age), intervention details (name, detailed regimen, duration, main technology, interactivity, and format), control regimen, outcomes, adverse events, and attrition rate.

### Quality Appraisal

The Effective Public Health Practice Project tool [48] was used to evaluate the methodological quality of the included studies via 6 aspects (selection bias, study design, confounders, blinding, data collection methods, and withdrawals and dropouts). Each aspect and the global rating were rated as “strong,” “moderate,” or “weak.” Two reviewers independently appraised the methodological quality, and any controversy was discussed until a consensus was reached.

### Data Analysis

A meta-analysis was performed when ≥2 studies with available data investigated the same outcome using similar effect measures; otherwise, the outcomes were presented narratively. For continuous variables, the mean difference (MD) with a 95% CI was applied only when the unit and instrument of measurement for an outcome were both the same across trials; otherwise, the standardized mean difference (SMD) with 95% CIs was selected [49]. For dichotomous variables, we used relative risks (RRs) with 95% CIs for point estimates. Heterogeneity between studies was estimated using *I^2^* test, and *I^2^* values of 25%, 50%, and 75% indicated low, medium, and high heterogeneity, respectively [49]. If *I^2^* was ≤50%, a fixed-effect model was adopted for analysis, whereas a random-effects model with more conservative estimates was used if *I^2^* was >50% [50]. Stata (version 12.0) was used for all the statistical calculations. A *P* value <.05 was considered statistically significant.

The primary outcome was analyzed using the following additional analyses. First, 3 subgroup analyses regarding intervention interactivity (interactive and noninteractive), format (personalized and nonpersonalized), and technology (mobile app and website) were performed, if possible, to detect the sources of heterogeneity and explore an optimal WBI regimen. Second, post hoc sensitivity analysis [51] was performed by including only RCTs to further identify whether the presence of different study designs was the potential source of heterogeneity. Finally, funnel plot and Egger test were conducted for outcomes involving ≥10 studies to identify publication bias [52].

## Results

### Study Selection

**Figure 1:**
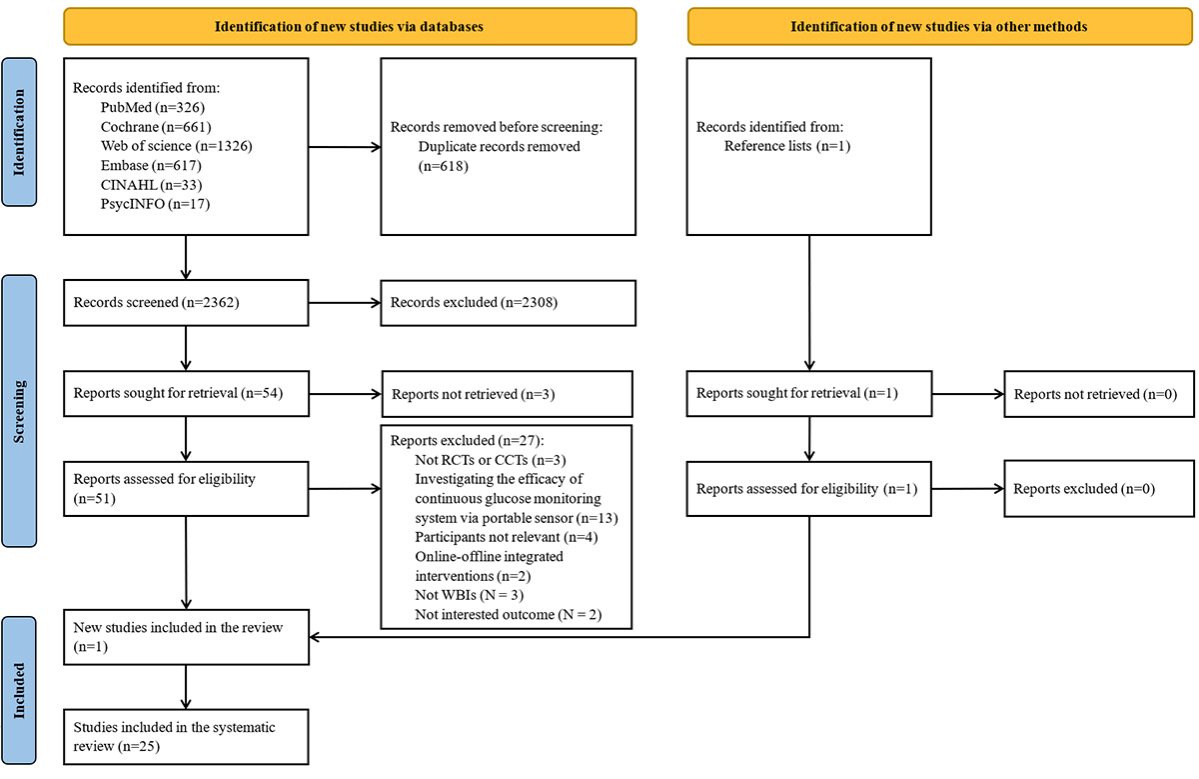
Flow diagram of search for and selection of relevant studies according to PRISMA (Preferred Reporting Items for Systematic Reviews and Meta-Analyses) 2020 guidelines. CCT: controlled clinical trial; RCT: randomized controlled trial; WBI: web-based intervention.

The database searches retrieved 2980 citations. After removing duplicates and screening the titles and abstracts, 1.71% (51/2980) of full-text articles were read. An additional article was identified through manual searching. Ultimately, 25 publications of 21 RCTs and CCTs met the eligibility criteria; 8 publications [25,30,40,42,43,53-55] were of 4 trials. The detailed screening process is illustrated in Figure 1.

### Methodological Quality Assessment

Overall, the studies were of good methodological quality in terms of global rating, with 28% (7/25) [28,31,38,42,53,54,56] rated as strong, 56% (14/25) [16,19,25-27,29,30,39-41,44,45,57,58] as moderate, and 16% (4/25) [15,43,55,59] as weak (Multimedia Appendix 3 [48]). With respect to selection bias, the participants in all trials were referred from clinics. The rate of agreement to participation was >80% in 72% (18/25) of studies [15,16,19,25-30,38,39,41,42,44,45,54,56,57] and between 60% and 79% in 12% (3/25) of studies [31,40,53], whereas 16% (4/25) of studies [43,55,58,59] did not report the details. Regarding study design, 76% (19/25) of publications were of RCTs [16,19,25,26,29-31,38-42,44,45,53,54,56,58,59], whereas 24% (6/25) were of CCTs [15,27,28,43,55,57]. In terms of confounders, no baseline difference was reported in 80% (20/25) of studies, whereas 20% (5/25) [15,40,45,53,57] had significant baseline differences, with 60% (3/5) [40,45,53] adopting an adjustment in statistical analyses. In addition, only 12% (3/25) of studies [31,53,56] applied a single-blinded method, whereas the others (22/25, 88%) were unable to blind participants or did not provide information on blinding. The instruments used for assessing outcomes were valid and reliable in all studies. In addition, except for 4% (1/25) of studies [27], the remaining 96% (24/25) of studies reported <20% dropouts.

### Study Characteristics

A summary of the characteristics of the included studies is provided in Table 1, and detailed characteristics are presented in Multimedia Appendix 4 [15,16,19,25-31,38-45,53-59]. A total of 25 publications with 2519 participants from 14 different countries were included. Except for 24% (6/25) of publications that were CCTs, the remaining 76% (19/25) were RCTs. The sample sizes of the studies ranged from 21 [39] to 340 [29]. The participants’ ages ranged from 18 to 45 years. The baseline gestational age was between 12 and 35 weeks. The diagnostic criteria for GDM were based on the International Association Diabetic Pregnancy Study Group, Carpenter and Coustan, Norwegian guidelines, or the integration of Carpenter and Coustan with additional risk factors for GDM, whereas 36% (9/25) of studies [19,25,27,41,43,44,54,55,57] did not report these details.

**Table 1:** Summary of the reviewed studies (N=25).

Study characteristic		Studies, n (%)
**Date of publication**		
	2018-2022 (in recent 5 years)	20 (80)
	2017 and before	5 (20)
**Group study site**		
	Australia	3 (12)
	Canada	1 (4)
	China	5 (20)
	Europe (Norway, Switzerland, Spain, or United Kingdom)	5 (20)
	South Korea	2 (8)
	Singapore	1 (4)
	United States	2 (8)
	West Asia (Saudi Arabia, Iran, Israel, or Turkey)	6 (24)
**Research design**		
	Randomized controlled trial	19 (76)
	Controlled clinical trial	6 (24)
**Gestational weeks at allocation**		
	Only included participants who were at ≥24 weeks of gestation	11 (44)
	Included participants who were at <24 and ≥24 weeks of gestation	14 (56)
**Diagnostic criteria of GDM^a^**		
	IADPSG^b^	10 (40)
	Carpenter and Coustan	3 (12)
	Norwegian guidelines	2 (8)
	Integration of Carpenter and Coustan and additional risk factor of GDM	1 (4)
	Not reported	9 (36)
**Sample size**		
	>100	13 (52)
	≤100	12 (48)
**Intervention duration**		
	>4 weeks	19 (76)
	≤4 weeks	6 (24)
**Main intervention technology**		
	Mobile app	16 (64)
	Website	8 (32)
	Mobile app or website	1 (4)
**Intervention interactivity**		
	Interactive	16 (64)
	Noninteractive	9 (36)
**Intervention format**		
	Nonpersonalized	14 (56)
	Personalized	11 (44)
**Control group**		
	Usual care	22 (88)
	One session of nutrition education	3 (12)
**Adverse event**		
	Not assessed	20 (80)
	Assessed	5 (20)
**Attrition rate (%)**		
	<20	24 (96)
	≥20	1 (4)

^a^GDM: gestational diabetes mellitus.

^b^IADPSG: International Association Diabetic Pregnancy Study Group.

It could be seen from the detailed intervention regimen that the main components of WBIs included diet advice, physical activity, glycemic level monitoring, disease education, medical treatments, emotional support, stress management, and peer support; the functionalities of WBIs could be summarized as spreading knowledge, documenting, uploading, downloading, tracking personal information, graphical progress, system alerts and reminders, and interactive communication. Among the 64% (16/25) of studies [15,19,26,29-31,38,39,41,42,44,45,56-59] with an interactive function, the frequency of feedback varied from daily [26,29,56] to biweekly [57]. The interactive personnel in 52% (13/25) of studies [15,19,26,29,31,38,39,41,45,56-59] were professionals, whereas both professionals and nonprofessionals (peer groups) were involved in 12% (3/25) of articles [30,42,44] on 2 trials. The format of WBIs was personalized in 44% (11/25) of studies [26-28,30,31,39,42,44,56,58,59] and nonpersonalized in 56% (14/25) of studies [15,16,19,25,29,38,40,41,43,45,53-55,57]. The main technology applied could be categorized as mobile apps (including disease-specific apps and social apps such as WeChat [Tencent Holdings Ltd] and WhatsApp [Meta Platforms, Inc]) and websites. None of the studies explicitly reported the intervention duration, which, based on the gestational weeks at the allocation and end points of intervention, varied from approximately 2 to 25 weeks. The participants in the control group received 1 session of nutrition education or usual care, whereas those in the intervention group received the same care as the control group and additional WBIs.

### The Effect of WBIs on Maternal Glycemic Control

#### Overview

**Figure 2:**
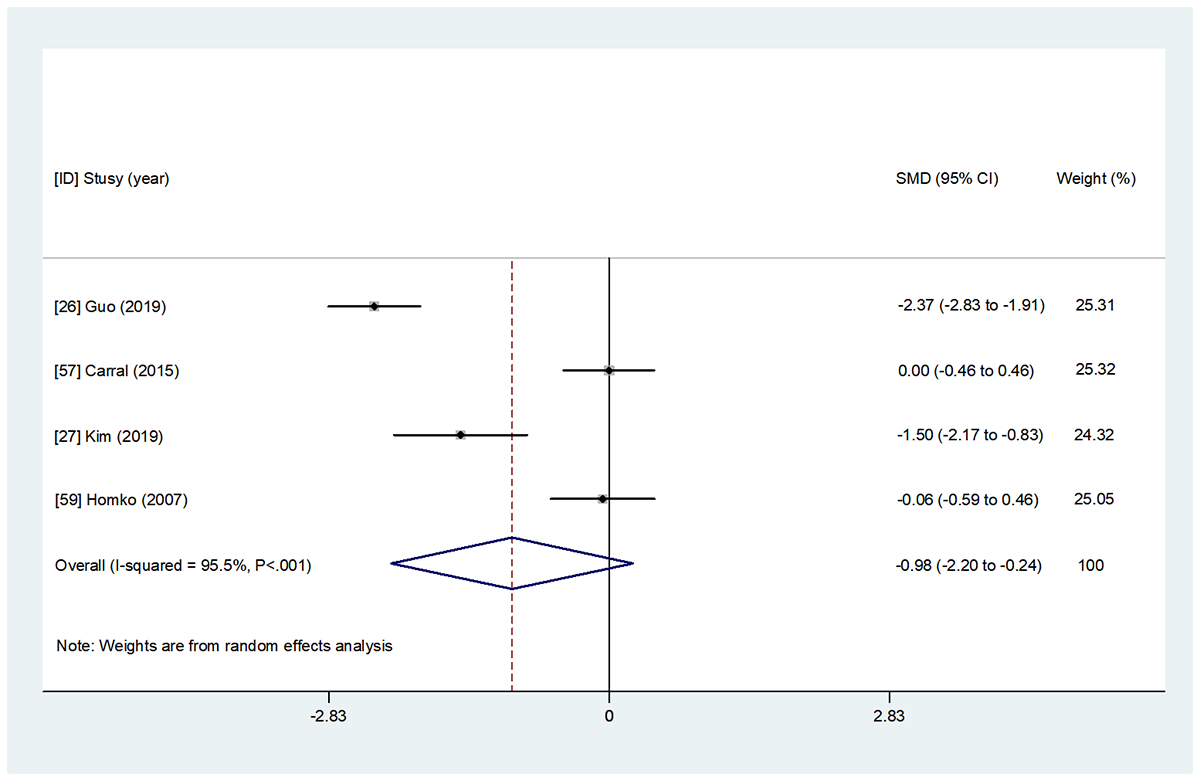
The effect of web-based interventions on glycated hemoglobin. SMD: standardized mean difference.

**Figure 3:**
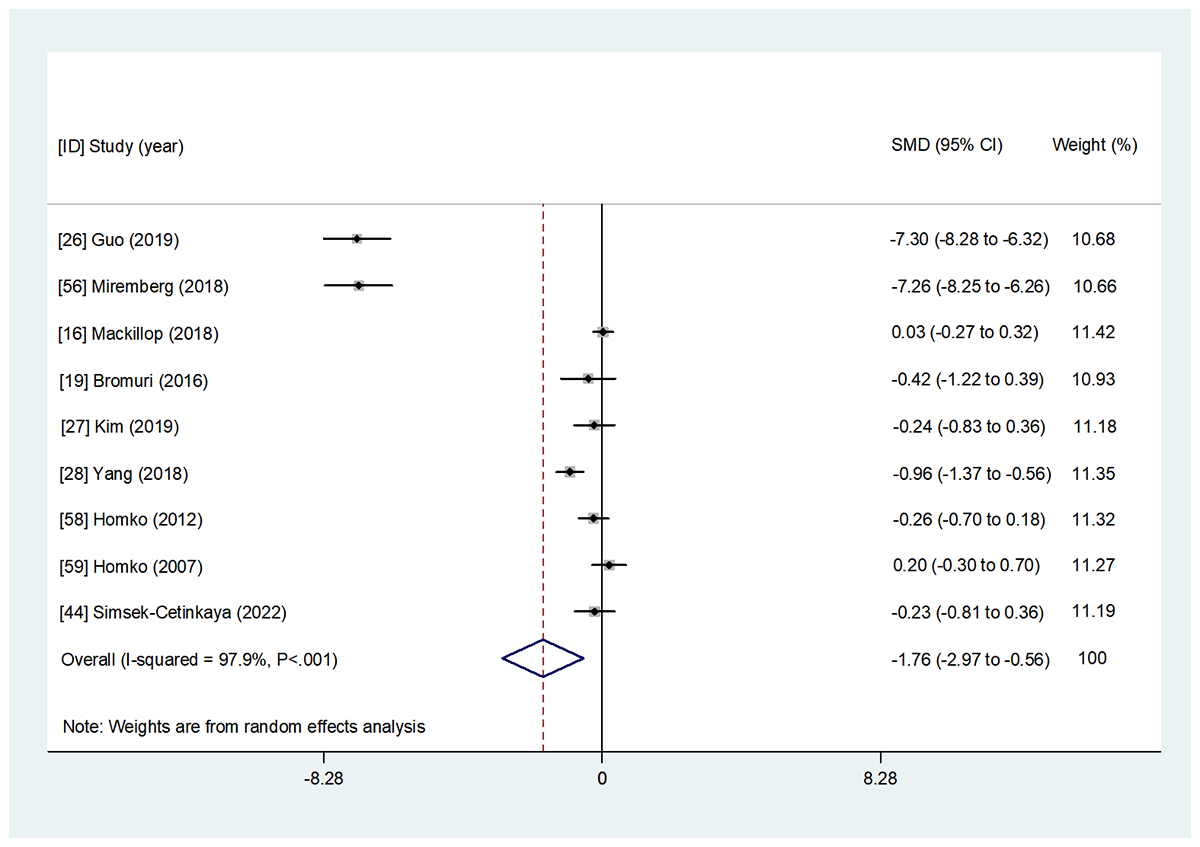
The effect of web-based interventions on fasting blood glucose. SMD: standardized mean difference.

**Figure 4:**
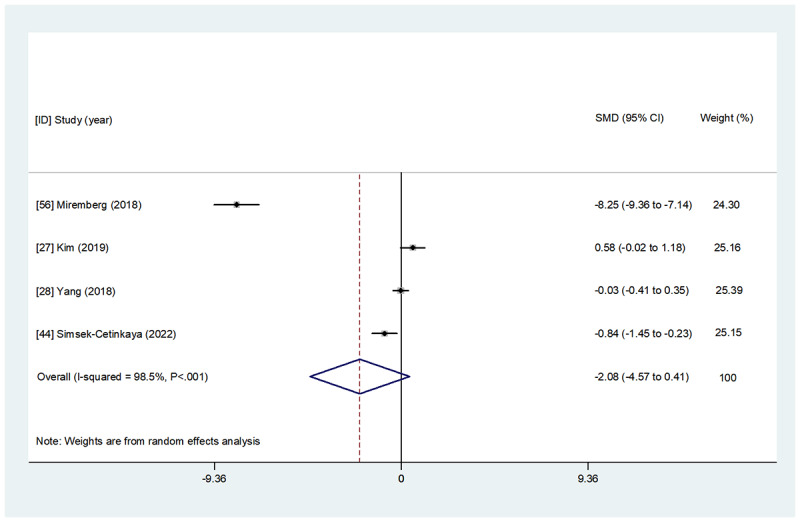
The effect of web-based interventions on 1-hour postprandial blood glucose. SMD: standardized mean difference.

**Figure 5:**
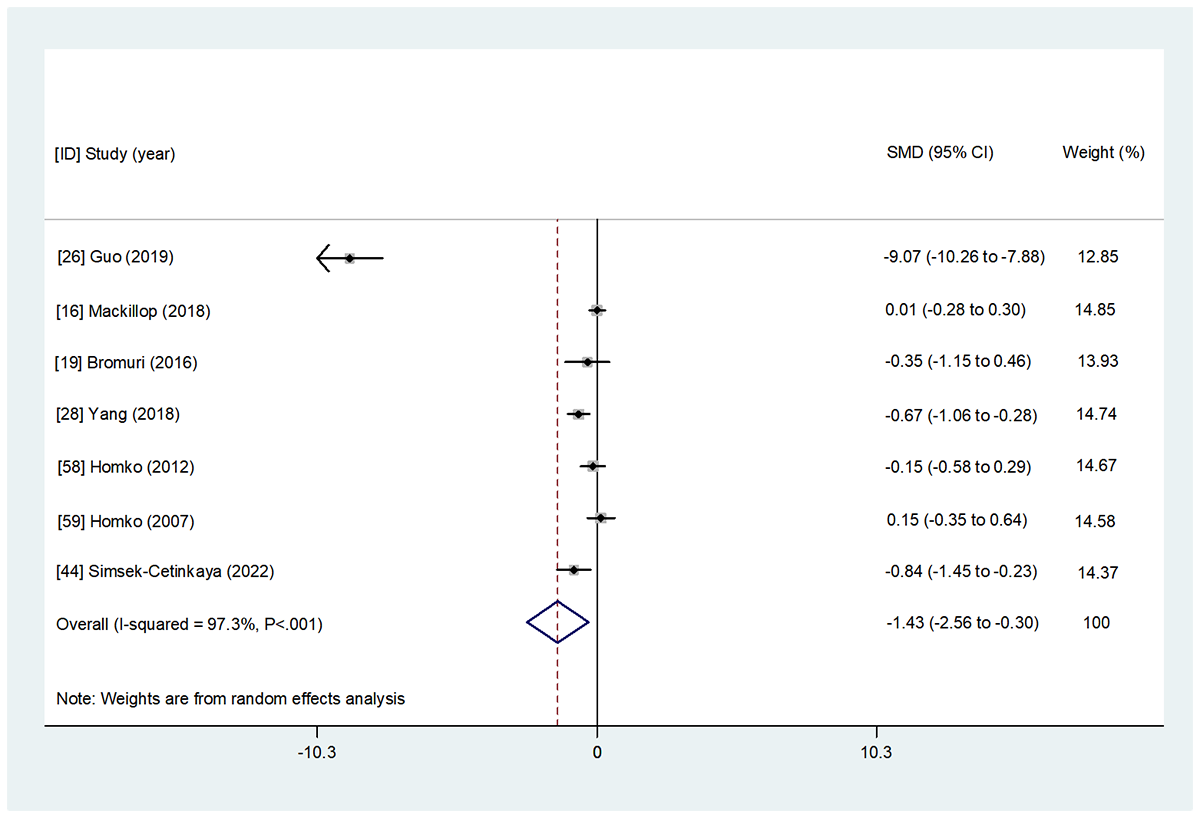
The effect of web-based interventions on 2-hour postprandial blood glucose. SMD: standardized mean difference.

Overall, 48% (12/25) of studies comprising 1042 participants examined the effect of WBIs on maternal glycemic control parameters (HbA_1c_, FBG, 1hBG, and 2hBG; Table 2 and Multimedia Appendix 4). Detailed results of the overall and subgroup meta-analyses for each indicator are presented in Figures 2-5 and Table 3. A summary of the results is presented in this section.

**Table 2:** Summary of the primary and secondary outcomes in the included studies (N=25).

Outcome		Studies that assessed this outcome, n (%)	Participants, n			References		Studies that were included in meta-analysis, n (%^a^)
			IG^b^	CG^c^				
**Primary outcome: maternal glycemic control**		12 (48)	533	509				
	HbA_1c_^d^	6 (24)	280	291	[16,26,27,38,57,59]		4 (67)	
	FBG^e^	11 (44)	503	462	[16,19,26-28,31,38,44,56,58,59]		9 (82)	
	1hBG^f^	4 (16)	162	154	[27,28,44,56]		4 (100)	
	2hBG^g^	9 (36)	421	380	[16,19,26,28,31,38,44,58,59]		7 (78)	
**Secondary outcome: maternal behavioral outcome**		13 (52)	892	882				
	Self-care behaviors—SMBG^h^	8 (32)	679	663	[16,19,26,29,31,42,45,56]		2 (25)	
	Self-care behaviors—overall self-care behaviors	2 (8)	64	64	[27,41]		0 (0)	
	Self-care behaviors—healthy diet behaviors	2 (8)	138	145	[40,44]		0 (0)	
	Self-care behaviors—physical activity	2 (8)	34	32	[39,44]		0 (0)	
**Secondary outcome: maternal cognitive and attitudinal outcome**		7 (28)	337	338				
	GDM^i^ knowledge	2 (8)	75	80	[25,44]		0 (0)	
	Risk perception of type 2 diabetes	1 (4)	45	45	[55]		0 (0)	
	Self-efficacy	2 (8)	79	74	[43,59]		0 (0)	
	Satisfaction with care	2 (8)	183	184	[15,16]		0 (0)	
**Secondary outcome: maternal mental health**		2 (8)	192	192				
	Depression	2 (8)	192	192	[27,29]		2 (100)	
	Anxiety	2 (8)	192	192	[27,29]		2 (100)	
**Secondary outcome: maternal and neonatal clinical outcome**		18 (72)	1163	1165				
	Insulin treatment rate	8 (32)	489	514	[19,29,44,53,56-59]		8 (100)	
	Oral antidiabetic drug treatment rate	4 (16)	196	198	[19,53,58,59]		4 (100)	
	Gestational weight gain	5 (20)	381	402	[16,26,29,39,57]		5 (100)	
	Induction of labor	2 (8)	172	181	[53,56]		2 (100)	
	Vaginal delivery	7 (28)	695	694	[16,26,28-30,53,56]		7 (100)	
	Normal vaginal delivery	6 (24)	549	555	[16,26,29,41,53,56]		6 (100)	
	Assisted vaginal delivery^j^	7 (28)	644	644	[15,16,26,28,29,53,56]		7 (100)	
	Cesarean delivery	15 (60)	1061	1056	[15,16,26,28-31,39,41,45,53,56-59]		15 (100)	
	Planned cesarean	6 (24)	584	568	[15,16,29,31,53,56]		6 (100)	
	Emergency cesarean	6 (24)	584	568	[15,16,29,31,53,56]		6 (100)	
	Gestational weeks at delivery	9 (36)	489	476	[15,16,26,31,39,56-59]		9 (100)	
	Premature delivery	10 (40)	753	771	[15,16,28-30,41,45,57-59]		10 (100)	
	Shoulder dystocia	4 (16)	304	303	[15,16,26,56]		4 (100)	
	Preeclampsia or gestational hypertension	8 (32)	580	592	[15,16,28,29,56-59]		8 (100)	
	Premature rupture of the membranes	6 (24)	400	393	[15,28,30,45,58,59]		6 (100)	
	Polyhydramnios	2 (8)	118	121	[45,56]		2 (100)	
	Macrosomia (≥4000 g)	9 (36)	787	771	[15,26,28-31,45,53,54]		9 (100)	
	Admission to the neonatal intensive care unit	9 (36)	691	660	[16,28,29,31,45,53,56,58,59]		9 (100)	
	Low birth weight (<2500 g)	3 (12)	265	275	[15,30,54]		3 (100)	
	Birth weight	11 (44)	709	695	[15,16,28,29,31,39,45,56-59]		11 (100)	
	Large for gestational age^k^	7 (28)	362	378	[15,16,39,56-59]		7 (100)	
	Small for gestational age^l^	3 (12)	130	154	[15,39,57]		3 (100)	
	Neonatal hypoglycemia	10 (40)	697	704	[15,16,26,28,29,45,56-59]		10 (100)	
	1-minute Apgar scores	2 (8)	72	65	[58,59]		2 (100)	
	5-minute Apgar scores	2 (8)	72	65	[58,59]		2 (100)	
	Neonatal jaundice or hyperbilirubinemia	6 (24)	457	441	[16,28,29,45,58,59]		6 (100)	
	Respiratory morbidity^m^	6 (24)	440	437	[15,29,45,56,58,59]		6 (100)	
	Composite neonatal complication^n^	3 (12)	264	259	[29,56,59]		3 (100)	
	Phototherapy	2 (8)	140	141	[15,56]		2 (100)	
	Neonatal death	3 (12)	288	291	[29,45,56]		3 (100)	
**Secondary outcome: medical service use and cost**		5 (20)	338	325				
	Frequency of medical service use	5 (20)	338	325	[15,16,26,31,57]		0 (0)	
	Medical service costs	3 (12)	244	218	[15,16,31]		0 (0)	

^a^Number of studies assessing the corresponding outcome was used as the denominator.

^b^IG: intervention group.

^c^CG: control group.

^d^HbA_1c_: glycated hemoglobin.

^e^FBG: fasting blood glucose.

^f^1hBG: 1-hour postprandial blood glucose.

^g^2hBG: 2-hour postprandial blood glucose.

^h^SMBG: self-monitoring of blood glucose.

^i^GDM: gestational diabetes mellitus.

^j^Assisted vaginal delivery included vacuum extraction, forceps delivery, and episiotomy.

^k^Large for gestational age: sex- and gestational age-adjusted birth weight >90^th^% of the population or customized standard.

^l^Small for gestational age: sex- and gestational age-adjusted birth weight <10^th^% of the population or customized standard.

^m^Respiratory morbidity included respiratory distress syndrome, transient tachypnea of the newborn, mechanical ventilation, and need for respiratory support.

^n^Composite neonatal complication was defined as the presence of ≥2 of the following: hypoglycemia of the newborn, respiratory morbidity, phototherapy, and neonatal death.

**Table 3:** Effect sizes and 95% CIs for web-based interventions on maternal glycemic control.

Outcome or subgroup title				References		Trials, n		Participants, n					SMD^a^ (IV^b^, random, 95% CI)			Overall effect, *P* value			Heterogeneity *I*^2^ value, %
								IG^c^		CG^d^									
**1. HbA_1c_^e^**				[26,27,57,59]		4		148		154		−0.98 (−2.199 to 0.239)			.12			95.5	
	**1.1 Subgroup analysis regarding intervention technology**					4									Subtotals only				
		1.1.1 Mobile app	[26]		1		64		60		−2.368 (−2.829 to −1.908)			<.001			N/A^f^		
		1.1.2 Website	[27,57,59]		3		84		94		−0.49 (−1.341 to 0.36)			.26			86.3		
	**1.2 Subgroup analysis regarding intervention interactivity**					4									Subtotals only				
		1.2.1 Interactive interventions	[26,57,59]		3		126		132		−0.813 (−2.375 to 0.749)			.31			96.9		
		1.2.2 Noninteractive interventions	[27]		1		22		22		−1.50 (−2.172 to −0.828)			<.001			N/A		
	**1.3 Subgroup analysis regarding intervention format**					4									Subtotals only				
		1.3.1 Personalized format	[26,27,59]		3		118		107		−1.312 (−2.756 to 0.131)			.08			95.2		
		1.3.2 Nonpersonalized format	[57]		1		30		47		0.00 (−0.458 to 0.458)			.99			N/A		
	1.4 Sensitivity analysis			[26,59]		2		96		85		−1.219 (−3.478 to 1.039)			.29			97.6	
**2. FBG^g^**				[16,19,26-28,44,56,58,59]		9		410		380		−1.764 (−2.972 to −0.557)			.004			97.9	
	**2.1 Subgroup analysis regarding intervention technology**					8									Subtotals only				
		2.1.1 Mobile app	[16,26,28,44,56]		5		302		277		−3.094 (−5.326 to −0.861)			.007			98.9		
		2.1.2 Website	[27,58,59]		3		96		91		−0.099 (−0.395 to 0.198)			.52			5.2		
	**2.2 Subgroup analysis regarding intervention interactivity**					9									Subtotals only				
		2.2.1 Interactive interventions	[19,26,44,56,58,59]		6		233		223		−2.51 (−4.716 to −0.303)			.03			98.6		
		2.2.2 Noninteractive interventions	[16,27,28]		3		177		157		−0.389 (−1.05 to 0.271)			.25			87		
	**2.3 Subgroup analysis regarding intervention format**					9									Subtotals only				
		2.3.1 Personalized format	[26-28,44,56-58]		7		300		283		−2.24 (−3.889 to −0.591)			.008			98.3		
		2.3.2 Nonpersonalized format	[16,19]		2		110		97		−0.026 (−0.304 to 0.251)			.85			.8		
	2.4 Sensitivity analysis			[16,19,26,44,56,58,59]		7		331		308		−2.124 (−3.771 to −0.478)			.01			98.4	
**3. 1hBG^h^**				[27,28,44,56]		4		162		154		−2.077 (−4.568 to 0.415)			.10			98.5	
	**3.1 Subgroup analysis regarding intervention technology**					4									Subtotals only				
		3.1.1 Mobile app	[28,44,56]		3		140		132		−2.993 (−6.523 to 0.538)			.10			98.9		
		3.1.2 Website	[27]		1		22		22		0.58 (−0.023 to 1.184)			.06			N/A		
	**3.2 Subgroup analysis regarding intervention interactivity**					4									Subtotals only				
		3.2.1 Interactive interventions	[44,56]		2		83		82		−4.529 (−11.792 to 2.733)			.22			99.2		
		3.2.2 Noninteractive interventions	[27,28]		2		79		72		0.23 (−0.358 to 0.817)			.44			64		
	3.3 Sensitivity analysis			[44,56]		2		83		82		−4.529 (−11.792 to 2.733)			.22			99.2	
**4. 2hBG^i^**				[16,19,26,28,44,58,59]		7		328		298		−1.433 (−2.561 to −0.304)			.01			97.3	
	**4.1 Subgroup analysis regarding intervention technology**					6									Subtotals only				
		4.1.1 Mobile app	[16,26,28,44]		4		242		217		−2.527 (−4.56 to −0.495)			.02			98.6		
		4.1.2 Website	[58,59]		2		74		69		−0.017 (−0.346 to 0.312)			.92			0		
	**4.2 Subgroup analysis regarding intervention interactivity**					7									Subtotals only				
		4.2.1 Interactive interventions	[19,26,44,58,59]		5		173		163		−1.979 (−3.987 to 0.029)			.05			98.1		
		4.2.2 Noninteractive interventions	[16,28]		2		155		135		−0.318 (−0.985 to 0.349)			.35			86.7		
	**4.3 Subgroup analysis regarding intervention format**					7									Subtotals only				
		4.3.1 Personalized format	[26,28,44,58,59]		5		218		201		−2.009 (−3.722 to −0.296)			.02			98.1		
		4.3.2 Nonpersonalized format	[16,19]		2		110		97		−0.032 (−0.305 to 0.242)			.82			0		
	4.4 Sensitivity analysis			[16,19,26,44,58,59]		6		271		248		−1.598 (−3.012 to −0.185)			.03			97.7	

^a^SMD: standardized mean difference.

^b^IV: inverse variance.

^c^IG: intervention group.

^d^CG: control group.

^e^HbA_1c_: glycated hemoglobin.

^f^N/A: not applicable.

^g^FBG: fasting blood glucose.

^h^1hBG: 1-hour postprandial blood glucose.

^i^2hBG: 2-hour postprandial blood glucose.

#### Overall Effect

##### Effect on HbA_1c_

A total of 24% (6/25) of studies [16,26,27,38,57,59] assessed the effect of WBIs on HbA_1c_, but only 33% (2/6) of studies [26,27] reported that WBIs significantly improved HbA_1c_ compared with the control group. A meta-analysis of 67% (4/6) of studies [26,27,57,59] with detailed data indicated no significant between-group difference in HbA_1c_ after the intervention (SMD=−0.98, 95% CI −2.199 to 0.239; *P*=.12).

##### Effect on FBG

The effect of WBIs on FBG was evaluated by 44% (11/25) of studies. Of them, 36% (4/11) of studies [19,26,28,56] showed that WBIs could significantly ameliorate FBG in the intervention group compared with the control group, whereas 64% (7/11) of studies [16,27,31,38,44,58,59] revealed no significant effect. A meta-analysis of 82% (9/11) of studies [16,19,26-28,44,56,58,59] with available data illustrated that WBIs were significantly associated with FBG amelioration compared with the control group after the intervention (SMD=−1.764, 95% CI −2.972 to −0.557; *P*=.004).

##### Effect on 1hBG

A total of 16% (4/25) of studies [27,28,44,56] with accessible data examined the effect of WBIs on 1hBG, but only 25% (1/4) of studies [56] found that WBIs elicited a significant amelioration in 1hBG compared with the control group. A meta-analysis of the (100%) studies demonstrated no significant between-group difference in 1hBG after the intervention (SMD=−2.077, 95% CI −4.568 to 0.415; *P*=.10).

##### Effect on 2hBG

A total of 36% (9/25) of studies estimated the effect of WBIs on 2hBG. Of them, 44% (4/9) [19,26,28,38] concluded that WBIs had a significant beneficial effect on 2hBG compared with the control group, whereas no significant improvement was reported in the remaining 56% (5/9) of studies [16,31,44,58,59]. A meta-analysis of 78% (7/9) of studies [16,19,26,28,44,58,59] with available data showed that after the intervention, the participants in the WBI group had a significantly lower 2hBG score than those in the control group (SMD=−1.433, 95% CI −2.561 to −0.304; *P*=.01).

#### Subgroup Analysis Regarding Intervention Interactivity

The outcome data of all glycemic control parameters of interest were divided into subgroups based on intervention interactivity. Eventually, the subgroup analysis revealed that the interactive subgroup had significant positive effects on FBG and had a tendency to be effective at improving 2hBG (*P*=.053), but no improvement was found in HbA_1c_; by contrast, the noninteractive subgroup could significantly improve HbA_1c_ (with only 1 study in the relevant subgroup) but not FBG and 2hBG. In addition, no significant effect on 1hBG was found in both subgroups. According to the existing evidence, interactive WBIs might exert amelioration on more glycemic control parameters than noninteractive WBIs.

#### Subgroup Analysis Regarding Intervention Format

Given that all primary studies assessing 1hBG implemented personalized WBIs, it was impossible to conduct subgroup analysis for this indicator based on the intervention format. Finally, the subgroup analysis showed that the personalized subgroup could significantly reduce FBG and 2hBG, but no improvement was found for these indicators in the nonpersonalized subgroup. Moreover, both the personalized and nonpersonalized subgroups showed no significant effect on HbA_1c_. Generally, personalized WBIs were more effective at improving glycemic control than nonpersonalized WBIs.

#### Subgroup Analysis Regarding Intervention Technology

As Bromuri et al [19] conducted WBIs via either a website or mobile app, it was difficult to categorize their study based on the intervention technology; therefore, the study was excluded from this round of subgroup analysis. Ultimately, the subgroup analysis demonstrated that the participants in the mobile app subgroup had significantly lower scores in terms of HbA_1c_, FBG, and 2hBG, whereas no significant amelioration was discovered for these indicators in the website subgroup. Moreover, there was no significant improvement in 1hBG in both subgroups. In general, mobile app–based interventions had a better effect on glycemic control than website-based interventions.

### The Effects of WBIs on Secondary Outcomes

#### Overview

Table 2 shows a summary of the outcomes, and the detailed results of each included study are presented in Multimedia Appendix 4. The results of the meta-analyses of secondary outcomes are shown in Table 4. The results of all the secondary outcomes are summarized in this section.

**Table 4:** Effect sizes and 95% CIs for the effects of web-based interventions on secondary outcomes

Outcome or subgroup title			References		Trials, n		Participants, n				Statistical method		Effect size		Overall effect, *P* value		Heterogeneity *I*^2^ value, %
							IG^a^		CG^b^								
**Maternal behavioral outcomes**																	
	Self-monitoring blood glucose compliance (%)^c^	[26,56]		2		124		120		MD^d^ (IV^e^, random, 95% CI)		15.856 (10.922 to 20.79)		<.001		84.1	
**Maternal mental health**																	
	Depression	[27,29]		2		192		192		SMD^f^ (IV, fixed, 95% CI)		−0.088 (−0.298 to 0.123)		.41		2.6	
	Anxiety	[27,29]		2		192		192		MD (IV, random, 95% CI)		−2.088 (−7.218 to 3.041)		.43		69.5	
**Maternal clinical outcomes**																	
	Insulin treatment rate	[19,29,44,53,56-59]		8		489		514		RR^g^ (M-H^h^, fixed, 95% CI)		0.795 (0.60 to 1.054)		.11		35.2	
	Oral antidiabetic drug treatment rate	[19,53,58,59]		4		196		198		RR (M-H, fixed, 95% CI)		0.738 (0.454 to 1.20)		.22		0	
	Gestational weight gain	[16,26,29,39,57]		5		381		402		SMD (IV, random, 95% CI)		−0.504 (−1.247 to 0.24)		.18		95.4	
	Induction of labor	[53,56]		2		172		181		RR (M-H, fixed, 95% CI)		1.004 (0.777 to 1.299)		.97		15.3	
	Vaginal delivery	[16,26,28-30,53,56]		7		695		694		RR (M-H, fixed, 95% CI)		1.041 (0.942 to 1.152)		.43		0	
	Normal vaginal delivery	[16,26,29,41,53,56]		6		549		555		RR (M-H, fixed, 95% CI)		1.16 (1.04 to 1.29)		.007		0	
	Assisted vaginal delivery	[15,16,26,28,29,53,56]		N=7		644		644		RR (M-H, fixed, 95% CI)		1.137 (0.834 to 1.55)		.42		0	
	Cesarean delivery	[15,16,26,28-31,39,41,45,53,56-59]		15		1061		1056		RR (M-H, fixed, 95% CI)		0.942 (0.834 to 1.065)		.34		15	
	Planned cesarean	[15,16,29,31,53,56]		6		584		568		RR (M-H, fixed, 95% CI)		1.005 (0.764 to 1.321)		.97		0	
	Emergency cesarean	[15,16,29,31,53,56]		6		584		568		RR (M-H, fixed, 95% CI)		0.623 (0.466 to 0.834)		.001		30	
	Gestational weeks at delivery (week)	[15,16,26,31,39,56-59]		9		489		476		MD (IV, random, 95% CI)		0.003 (−0.280 to 0.287)		.98		53.2	
	Premature delivery	[15,16,28-30,41,45,57-59]		10		753		771		RR (M-H, fixed, 95% CI)		0.827 (0.589 to 1.161)		.27		20.5	
	Shoulder dystocia	[15,16,26,56]		4		304		303		RR (M-H, fixed, 95% CI)		3.99 (0.45 to 35.397)		.21		0	
	Preeclampsia or gestational hypertension	[15,16,28,29,56-59]		8		580		592		RR (M-H, fixed, 95% CI)		0.957 (0.574 to 1.594)		.87		0	
	Premature rupture of the membranes	[15,28,30,45,58,59]		6		400		393		RR (M-H, fixed, 95% CI)		0.824 (0.572 to 1.186)		.30		0	
	Polyhydramnios	[45,56]		2		118		121		RR (M-H, random, 95% CI)		0.55 (0.042 to 7.18)		.65		58.1	
**Neonatal clinical outcomes**																	
	Macrosomia (≥4000 g)	[15,26,28-31,45,53,54]		9		787		771		RR (M-H, fixed, 95% CI)		0.801 (0.548 to 1.17)		.25		0	
	Admission to the neonatal intensive care unit	[16,28,29,31,45,53,56,58,59]		9		691		660		RR (M-H, fixed, 95% CI)		0.754 (0.58 to 0.979)		.03		0	
	Low birth weight (<2500 g)	[15,30,54]		3		265		275		RR (M-H, fixed, 95% CI)		1.381 (0.572 to 3.333)		.47		43.7	
	Birth weight (g)	[15,16,28,29,31,39,45,56-59]		11		709		695		MD (IV, fixed, 95% CI)		8.35 (−41.181 to 57.882)		.74		0	
	Large for gestational age	[15,16,39,56-59]		7		362		378		RR (M-H, fixed, 95% CI)		1.299 (0.898 to 1.878)		.17		0	
	Small for gestational age	[15,39,57]		3		130		154		RR (M-H, fixed, 95% CI)		1.231 (0.616 to 2.461)		.56		0	
	Neonatal hypoglycemia	[15,16,26,28,29,45,56-59]		10		697		704		RR (M-H, fixed, 95% CI)		0.959 (0.732 to 1.257)		.76		0	
	1-minute Apgar scores	[58,59]		2		72		65		MD (IV, fixed, 95% CI)		−0.292 (−0.872 to 0.289)		.33		14.3	
	5-minute Apgar scores	[58,59]		2		72		65		MD (IV, fixed, 95% CI)		−0.003 (−0.181 to 0.174)		.97		0	
	Neonatal jaundice or hyperbilirubinemia	[16,28,29,45,58,59]		6		457		441		RR (M-H, fixed, 95% CI)		0.904 (0.668 to 1.225)		.52		0	
	Respiratory morbidity	[15,29,45,56,58,59]		6		440		437		RR (M-H, fixed, 95% CI)		0.842 (0.578 to 1.227)		.37		0	
	Composite neonatal complication	[29,56,59]		3		264		259		RR (M-H, fixed, 95% CI)		0.78 (0.63 to 0.96)		.02		36	
	Phototherapy	[15,56]		2		140		141		RR (M-H, fixed, 95% CI)		0.568 (0.216 to 1.496)		.25		0	
	Neonatal death	[29,45,56]		N=3		288		291		RR (M-H, fixed, 95% CI)		0.262 (0.030 to 2.333)		.23		0	

^a^IG: intervention group.

^b^CG: control group.

^c^Self-monitoring blood glucose compliance (%) = actual blood glucose measurements / instructed measurements × 100.

^d^MD: mean difference.

^e^IV: inverse variance.

^f^SMD: standardized mean difference.

^g^RR: risk ratio.

^h^M-H: mantel-haenszel.

#### Maternal Behavioral Outcomes

Maternal behavioral outcomes referred to the self-care behaviors related to GDM, mainly including SMBG, healthy diet behaviors, and physical activity, which were assessed in 52% (13/25) of studies involving 1774 participants. Specifically, 32% (8/25) of studies [16,19,26,29,31,42,45,56] investigated the effect of WBIs on compliance with the SMBG using various outcome reporting forms. Of them, 62% (5/8) of studies [16,19,26,45,56] indicated that WBIs significantly increased compliance in the intervention group compared with the control group, whereas 38% (3/8) of studies [29,31,42] showed no between-group significance. A meta-analysis of 25% (2/8) of studies [26,56] with detailed data on compliance (%) further elicited a positive effect, favoring WBIs (MD=15.856, 95% CI 10.922-20.79; *P*<.001). The effect of WBIs on overall self-care behaviors [27,41], physical activity [39,44], and healthy diet behaviors [40,44] were assessed in comparison with the control group in 15% (2/13) of studies, respectively. However, given that different outcome parameters were reported and detailed data were not provided in some of the included studies, it was impossible to conduct quantitative syntheses. All outcomes had mixed results, that is, only half of these studies demonstrated that WBIs significantly improved participants’ overall self-care behaviors (1/2, 50%), physical activity (1/2, 50%), and healthy diet behaviors (1/2, 50%).

#### Maternal Cognitive and Attitudinal Outcomes

The cognitive and attitudinal outcomes of interest included knowledge of the disease, risk perception of the disease, self-efficacy, and satisfaction with care in pregnant women with GDM. A total of 28% (7/25) of studies comprising 675 participants evaluated maternal cognitive and attitudinal outcomes through 4 parameters. Considering the limited number of primary studies for each outcome, different reporting parameters, and the lack of detailed data in some primary studies, a narrative method was used to synthesize the results. All relevant studies reported significant positive postintervention effects on GDM knowledge (2/2, 100%) [25,44], risk perception of type 2 diabetes (1/1, 100%) [55], self-efficacy (2/2, 100%) [43,59], and satisfaction with care (2/2, 100%) [15,16] in the intervention group compared with the control group.

#### Maternal Mental Health

Two studies [27,29] with available data comprising 384 participants evaluated the effects of WBIs both on depression and anxiety in pregnant women with GDM. Meta-analyses revealed that WBIs did not significantly alleviate depression (SMD=−0.088, 95% CI −0.298 to 0.123; *P*=.41) or anxiety (MD=−2.088, 95% CI −7.218 to 3.041; *P*=.43) when compared with the control group.

#### Maternal and Neonatal Clinical Outcomes

A total of 32% (8/25) of studies involving 2328 participants investigated the effect of WBIs on maternal and neonatal clinical outcomes, and all relevant studies provided detailed data. Meta-analyses showed that WBIs exhibited significant positive effects on normal vaginal delivery (RR=1.16, 95% CI 1.04-1.29; *P*=.007), emergency cesarean (RR=0.623, 95% CI 0.466-0.834; *P*=.001), admission to the neonatal intensive care unit (RR=0.754, 95% CI 0.58-0.979; *P*=.03), and composite neonatal complications (RR=0.78, 95% CI 0.63-0.96; *P*=.02) in the intervention group compared with the control group, whereas the effects on all other clinical outcomes were not significant (*P*>.05).

#### Medical Service Use and Costs

A total of 20% (5/25) of studies [15,16,26,31,57] containing 663 participants conducted economic and health service use analyses. Owing to the presence of various outcome reporting formats, the results were synthesized narratively. Specifically, all these studies [15,16,26,31,57] evaluated the effect of WBIs on the frequency of medical service use in the WBI group compared with the control group. Of them, 60% (3/5) of studies [15,26,57] found a significant improvement in medical service use in the WBI group, whereas the remaining 40% (2/5) of studies [16,31] reported no between-group difference. In addition, 60% (3/5) of studies [15,16,31] assessed the effects of WBIs on medical service costs among pregnant women with GDM, but mixed results were generated. Overall, 33% (1/3) of studies [15] found a significant positive postintervention effect compared with the control group, whereas 67% (2/3) of studies [16,31] reported no between-group difference.

### Sensitivity Analysis and Publication Bias for Maternal Glycemic Control

In the post hoc sensitivity analysis, the heterogeneity of HbA_1c_, FBG, 1HBG, and 2hBG did not decrease, and the effects of the meta-analyses remained unchanged (Table 3). In addition, for each parameter of maternal glycemic control, there were <10 primary studies with available data; therefore, publication bias assessment was not necessary.

## Discussion

### Summary and Interpretation of Findings

To the best of our knowledge, this is the first systematic review and meta-analysis based on all the existing RCTs and CCTs to investigate the all-round efficacy of WBIs in pregnant women with GDM. Meta-analyses of maternal glycemic control parameters indicated that compared with the control group, WBIs significantly ameliorated FBG and 2hBG but not HbA_1c_ and 1hBG. Other beneficial effects of WBIs in pregnant women with GDM were also discovered, including improved compliance with SMBG, maternal cognitive and attitudinal outcomes, medical service use, and normal vaginal delivery as well as reduced emergency cesarean, admission to the neonatal intensive care unit, and composite neonatal complications. However, the effectiveness of WBIs on other secondary outcomes was nonsignificant or inconclusive owing to insufficient evidence. Some studies assessed the adverse events of WBIs and reported that none occurred [31,40,45,53] or that no between-group difference was found [19], indicating that WBIs were relatively safe.

As for maternal glycemic control, on the one hand, this review demonstrated that WBIs could significantly reduce FBG and 2hBG in pregnant women with GDM, which was supported by 2 previous meta-analyses [34,35]. However, the meta-analysis by Li et al [23] revealed a significant reduction in the mean (1-h and 2-h) postprandial blood glucose but not in FBG after telemedicine interventions in the women with GDM compared with those in the control group. This inconsistency might have resulted from the different primary studies being analyzed among reviews; specifically, Li et al [23] focused on telemedicine-based lifestyle interventions and drew the conclusion about FBG based only on the result of a meta-analysis of 5 RCTs. The significant positive effects of WBIs on FBG and 2hBG could be explained as follows. First, WBIs make GDM management more continuous, comprehensive, and timely in a limited time window [30], which is helpful in enhancing patients’ self-awareness and confidence in managing GDM and improving treatment adherence [16,53,60]. The discovery of this review that participants in the WBIs group had significantly better compliance with SMBG confirmed this point of view. Second, diet and physical activity were introduced as intervention components in some included studies [25,28,29], which could provide additional benefits for glycemic control and improve intervention efficacy [13]. On the other hand, the effects of WBIs on HbA_1c_ and 1hBG were found to be insignificant, which is consistent with the findings of relevant reviews [23,32,36]. The possible interpretation of the nonsignificant results might be as follows:

1. Insufficient intervention duration and a less sensitive indicator: HbA_1c_ is known as a 3-month mean measure of glycemic control, whereas the intervention duration for some participants might not yet have reached 3 months [59]. Beyond that, HbA_1c_ is less sensitive in pregnant women owing to iron deficiency and increased turnover of red blood cells during pregnancy [61].
2. Bias of self-reported data: in the included studies, 1hBG as a self-reporting indicator might not have been monitored at the required timing or recorded as correctly [31], which may have had a significant impact on the accuracy of effect assessment.
3. Limited number of original trials: given that only 4 trials comprising 302 participants and 4 trials comprising 316 participants were included in the meta-analyses of HbA_1c_ and 1hBG, respectively, the pooled data might be underpowered to detect a statistically significant difference, which needs to be verified in more studies.

Notably, the findings of the 3 subgroup analyses on the primary outcome provided insights for developing a scientific WBI regimen. Subgroup analyses regarding intervention interactivity and format indicated that interactive and personalized subgroups had more beneficial effects on glycemic control than noninteractive and nonpersonalized subgroups, respectively. A plausible explanation for these better effects is that interactive and personalized interventions can increase reciprocal communications between health practitioners and patients and enable the former to understand the latter’s unmet needs dynamically, thereby providing them with targeted advice and care based on their physical conditions, characteristics of symptoms, abilities, values, beliefs, and likes and dislikes, which can ultimately be helpful in sustaining high user engagement, enhancing satisfaction with care, and maximizing interventional effects. Interestingly, we noticed that peer support had been integrated into WBIs in some studies [30,42,44], that is, bidirectional patient-patient interaction was allowed during the intervention, which has been demonstrated to be an effective approach for increasing patients’ self-confidence in disease control [30] and is worthy of being recommended. In addition, subgroup analysis of intervention technology showed that the mobile app subgroup had a better effect on glycemic control than the website subgroup. The most probable reason for this finding is that mobile apps are easily accessible in daily life and enable participants to receive interventions in fragmented time, whereas websites are usually browsed via computers and are not as convenient as mobile apps. Accordingly, we recommend developing personalized, interactive, and app-delivered interventions to manage GDM more effectively.

As for the secondary outcomes, this review found significant improvements in the compliance with SMBG, normal vaginal delivery, emergency cesarean, admission to the neonatal intensive care unit, and composite neonatal complications in the WBI group compared with the control group. Nevertheless, we failed to demonstrate significant between-group differences in other maternal and neonatal clinical outcomes or maternal mental health, which were approximately consistent with the findings of relevant reviews [23,32,36,37]. The effects might have been insignificant because of the fact that the intervention duration was too short to bring measurable changes [29,53,59], and maternal and neonatal clinical outcomes might be more influenced by local medical treatment levels than directly by the intervention itself [37]. It is noteworthy that despite the lack of definitive conclusions owing to the inability to quantitatively synthesize the results, all the cognitive and attitudinal outcomes (including GDM knowledge, risk perception of type 2 diabetes, self-efficacy, and satisfaction with care) of the participants in the WBI group were ameliorated compared with those of the participants in the control group. This was inspiring because improvements in cognition and attitude are known as the prerequisites for the implementation of healthy behaviors [62], whereas the latter can directly induce better health outcomes. Moreover, WBIs also showed a beneficial effect on medical service use but not on medical service costs based on the limited existing evidence. Nevertheless, what should be emphasized is that a cost-effectiveness analysis of WBIs is extremely important for future research, as one of the main purposes of WBIs is to relieve the shortage of medical resources and reducing costs.

### Implications for Future Research and Clinical Practice

Several priorities for future research and practice were identified. First and foremost, we noticed that only 1 trial [29] developed an intervention program based on a theoretical model. Given that theories can explain the underlying mechanisms and determinants of behavior changes and help select the most beneficial method for implementing behavior changes for people [63], future studies should consider the theoretical basis of WBIs. Besides, although blinding participants is usually impossible in WBIs, training staff in the aspects of intervention implementation and data collection, as well as conducting randomization and allocation hiding adequately, are helpful to improve the methodological quality of studies. Moreover, glycated albumin was evaluated in 4% (1/25) of the included studies [27], which can reflect glycemic control levels during pregnancy more sensitively than HbA_1c_ [64] and deserves to be recommended as an outcome indicator in more trials. Furthermore, there is an urgent need to develop user-friendly WBIs by resolving technical issues, improving operability, and incorporating gamification elements (such as progress bars and leaderboards), thereby enhancing participants’ interests and compliance to achieve better effects. In addition, it is essential to construct a standardized WBI evaluation framework for conducting comprehensive cost analyses that include both direct and indirect medical costs (eg, parking, transportation, and work absenteeism). In addition, it has been demonstrated that earlier implementation of WBIs could lead to more beneficial effects [30]. Consequently, we suggest starting WBIs immediately after the diagnosis of GDM or even providing customized WBIs for women with risk factors for GDM (such as obesity) during the planning stage or at the start of a pregnancy. Moreover, clinical practitioners should spare no effort to explore how to integrate WBIs into existing health care systems more appropriately, thereby improving the efficiency of medical services. Finally, a secure internet environment should be established to protect participants’ privacy before the widespread application of WBIs.

### Strengths and Limitations

This is the first systematic review and meta-analysis to comprehensively summarize the effects of WBIs in pregnant women with GDM. Our findings were based on relatively recent evidence, as 64% (n=16) of the included studies were published in the past 4 years, which were the times of the COVID-19 pandemic. WBIs are helpful in significantly reducing the risk of COVID-19 infection because of the lack of face-to-face contact [35], which may be a critical reason for the public’s increased attention, preferences, and use of WBIs in the past 4 years. Concomitantly, WBIs have opened up an encouraging and novel direction for the reform of the health care delivery model in times of social distancing and isolation measures, as well as other situations involving reduced access to resources or low levels of mobility. Moreover, 3 subgroup analyses regarding intervention format, interactivity, and technology were performed in this review, which provided useful information for developing an optimal WBIs regimen.

Admittedly, this review has several limitations. First, 56% (n=14) of the included studies were conducted in developed countries with high access to the internet, which might limit the dissemination of the findings to marginalized groups in developing regions. Accordingly, extensive prospective studies on this topic in different countries are required before the widespread dissemination of WBIs. Second, the nature of WBIs makes a double-blind design impossible, which might cause a Hawthorne effect [65] and exaggerate clinical improvements in some outcomes. Fortunately, the primary outcome of this review—maternal glycemic control—was relatively less likely to be affected by insufficient implementation of blinding because of its objectivity. Third, significant heterogeneity in the overall and most subgroup analyses weakened the reliability of the findings. Possible explanations for the origins of the heterogeneity are as follows: (1) the subgroup analysis based on the intervention format indicated that the personalized format might be the main reason for heterogeneity, as the heterogeneity disappeared (*I^2^*=0) in the nonpersonalized subgroups for all glycemic control parameters (Table 3), and (2) the differences in the diagnostic criteria for GDM, gestational weeks at allocation, and intervention duration might have also caused some heterogeneity. However, the difference in study design (RCT and CCT) was not the source of heterogeneity according to the results of the sensitivity analysis. Hence, future studies should adequately elaborate on the details of personalized WBIs and reach a consensus on the best GDM diagnostic criteria, the optimal duration of WBIs, and the appropriate gestational week for the initiation of WBIs so as to decrease the heterogeneity and increase the reproducibility of results. Fourth, some relevant trials might have been missed because only studies published in English were included. Given these limitations, the findings of this review should be interpreted with caution.

### Conclusions

In summary, WBIs were effective in ameliorating FBG and 2hBG and could also significantly enhance compliance with SMBG; increase the chance of normal vaginal delivery; and decrease the chance of emergency cesarean, admission to the neonatal intensive care unit, and composite neonatal complications in pregnant women with GDM. Moreover, WBIs were possibly effective in improving GDM knowledge, risk perception of disease, self-efficacy, satisfaction with care, and medical service use, but the evidence for this lacks certainty. Nevertheless, the effectiveness of WBIs in other outcomes of interest is insignificant or uncertain. Personalized, interactive, and mobile app–delivered WBIs are especially worthy of being implemented. However, owing to the high heterogeneity and limited number of original studies for most outcomes, our findings should be interpreted with caution. Further well-designed and sufficiently powered RCTs should be conducted to provide robust evidence for future practice.

## Appendix

Multimedia Appendix 1 PRISMA (Preferred Reporting Items for Systematic Reviews and Meta-Analyses) checklist.

Multimedia Appendix 2 Literature search strategy.

Multimedia Appendix 3 Assessment for the methodological quality.

Multimedia Appendix 4 Characteristics of the studies included in this systematic review.

## Abbreviations

1hBG: 1-hour postprandial blood glucose
2hBG: 2-hour postprandial blood glucose
CCT: controlled clinical trial
FBG: fasting blood glucose
GDM: gestational diabetes mellitus
HbA_1c_: glycated hemoglobin
MD: mean difference
PRISMA: Preferred Reporting Items for Systematic Reviews and Meta-Analyses
RCT: randomized controlled trial
RR: relative risk
SMBG: self-monitoring of blood glucose
SMD: standardized mean difference
WBI: web-based intervention
