# Supplementary material for: Web-Based Interventions for Pregnant Women With Gestational Diabetes Mellitus: Systematic Review and Meta-analysis
Source: J Med Internet Res. 2023 Jan 19;25:e36922. doi: 10.2196/36922 (PMC9896357; doi:10.2196/36922)
Supplement: Multimedia Appendix 2 [file jmir_v25i1e36922_app2.docx]

Literature search strategy.

| Electronic database  (From each database inception to Nov. 19th 2022) | Search terms | Number  of articles |
| --- | --- | --- |
| PubMed | "Diabetes, Gestational"[Mesh] OR ((((((((((Diabetes, Pregnancy-Induced[Title/Abstract]) OR (Diabetes, Pregnancy Induced[Title/Abstract])) OR (Diabetes Mellitus, Gestational[Title/Abstract])) OR (Diabetes, Gestational[Title/Abstract])) OR (diabetes in pregnancy[Title/Abstract])) OR (Gestational Diabetes[Title/Abstract])) OR (Gestational Diabetes Mellitus[Title/Abstract])) OR (GDM[Title/Abstract])) OR (maternal diabetes[Title/Abstract])) OR (Pregnancy-Induced Diabetes[Title/Abstract])) OR (pregnancy diabetes mellitus[Title/Abstract])  AND (((((((("Mobile Applications"[Mesh]) OR "Telemedicine"[Mesh]) OR "Internet"[Mesh]) OR "Computers"[Mesh]) OR "Telecommunications"[Mesh]) OR "Online Systems"[Mesh]) OR "Software"[Mesh]) OR "Wireless Technology"[Mesh]) OR "Cell Phone"[Mesh] OR ((((((((((((((((((((((((((((((((((((((((((((((((((((((((((((((((((((((((((((app[Title/Abstract]) OR (apps[Title/Abstract])) OR (application[Title/Abstract])) OR (applications[Title/Abstract])) OR (ipad[Title/Abstract])) OR (blog[Title/Abstract])) OR (blogging[Title/Abstract])) OR (computer[Title/Abstract])) OR (computer interface[Title/Abstract])) OR (cell phones[Title/Abstract])) OR (cell phone[Title/Abstract])) OR (cellular phone[Title/Abstract])) OR (digital[Title/Abstract])) OR (digital health[Title/Abstract])) OR (digital-health[Title/Abstract])) OR (ehealth[Title/Abstract])) OR (e-health[Title/Abstract])) OR (e-mail[Title/Abstract])) OR (electronic[Title/Abstract])) OR (E-learning[Title/Abstract])) OR (Facebook[Title/Abstract])) OR (health, mobile[Title/Abstract])) OR (health technolog[Title/Abstract])) OR (health app[Title/Abstract])) OR (Internet[Title/Abstract])) OR (Internet forum[Title/Abstract])) OR (iphone[Title/Abstract])) OR (i phone[Title/Abstract])) OR (i-phone[Title/Abstract])) OR (ipad[Title/Abstract])) OR (i pad[Title/Abstract])) OR (i-pad[Title/Abstract])) OR (laptop[Title/Abstract])) OR (linkedin[Title/Abstract])) OR (mobile[Title/Abstract])) OR (mobile application[Title/Abstract])) OR (mobile apps[Title/Abstract])) OR (mobile app[Title/Abstract])) OR (mobile phone[Title/Abstract])) OR (mobile phones[Title/Abstract])) OR (mhealth[Title/Abstract])) OR (m-health[Title/Abstract])) OR (mobile health[Title/Abstract])) OR (mobile electronic device[Title/Abstract])) OR (mobile technolog[Title/Abstract])) OR (mobile communication[Title/Abstract])) OR (mobile computing[Title/Abstract])) OR (network[Title/Abstract])) OR (online[Title/Abstract])) OR (online intervention[Title/Abstract])) OR (online interventions[Title/Abstract])) OR (platform[Title/Abstract])) OR (personal computer[Title/Abstract])) OR (personal digital assistant[Title/Abstract])) OR (QQ[Title/Abstract])) OR (remote[Title/Abstract])) OR (smartphone[Title/Abstract])) OR (smart phone[Title/Abstract])) OR (social media[Title/Abstract])) OR (social networking[Title/Abstract])) OR (telehealth[Title/Abstract])) OR (tele-health[Title/Abstract])) OR (telephone[Title/Abstract])) OR (telemedicine[Title/Abstract])) OR (tele-medicine[Title/Abstract])) OR (tele-care[Title/Abstract])) OR (telecare[Title/Abstract])) OR (telecommunication[Title/Abstract])) OR (telemonitor[Title/Abstract])) OR (tele-monitor[Title/Abstract])) OR (telemonitoring[Title/Abstract])) OR (twitter[Title/Abstract])) OR (web[Title/Abstract])) OR (web-based[Title/Abstract])) OR (website[Title/Abstract])) OR (wireless[Title/Abstract])) OR (WeChat[Title/Abstract])  AND ((("Randomized Controlled Trial" [Publication Type]) OR "Randomized Controlled Trials as Topic"[Mesh]) OR "Controlled Clinical Trial" [Publication Type]) OR "Controlled Clinical Trials as Topic"[Mesh] OR Clinical Trials, Randomized[Title/Abstract] OR Trials, Randomized Clinical[Title/Abstract] OR Controlled Clinical Trials, Randomized[Title/Abstract] OR RCT[Title/Abstract] OR Clinical Trials[Title/Abstract] OR Controlled Clinical Trials[Title/Abstract] OR CCT[Title/Abstract] | 326 |
| Embase | #1 'pregnancy diabetes mellitus'/exp OR 'diabetes, pregnancy-induced':ab,ti OR 'diabetes, pregnancy induced':ab,ti OR 'diabetes mellitus, gestational':ab,ti OR 'diabetes, gestational':ab,ti OR 'diabetes in pregnancy':ab,ti OR 'gestational diabetes':ab,ti OR 'gestational diabetes mellitus':ab,ti OR gdm:ab,ti OR 'maternal diabetes':ab,ti OR 'pregnancy-induced diabetes':ab,ti OR 'pregnancy diabetes mellitus':ab,ti  #2 'mobile application'/exp OR 'telemedicine'/exp OR 'internet'/exp OR 'computer'/exp OR 'telecommunication'/exp OR 'online system'/exp OR 'software'/exp OR 'wireless communication'/exp OR 'mobile phone'/exp OR app:ab,ti OR apps:ab,ti OR application:ab,ti OR applications:ab,ti OR blog:ab,ti OR blogging:ab,ti OR computer:ab,ti OR 'computer interface':ab,ti OR 'cell phones':ab,ti OR 'cell phone':ab,ti OR 'cellular phone':ab,ti OR digital:ab,ti OR 'digital health':ab,ti OR 'digital-health':ab,ti OR ehealth:ab,ti OR 'e-health':ab,ti OR 'e-mail':ab,ti OR electronic:ab,ti OR 'e-learning':ab,ti OR facebook:ab,ti OR 'health, mobile':ab,ti OR 'health technolog':ab,ti OR 'health app':ab,ti OR internet:ab,ti OR 'internet forum':ab,ti OR iphone:ab,ti OR 'i phone':ab,ti OR 'i-phone':ab,ti OR ipad:ab,ti OR 'i pad':ab,ti OR 'i-pad':ab,ti OR laptop:ab,ti OR linkedin:ab,ti OR mobile:ab,ti OR 'mobile application':ab,ti OR 'mobile apps':ab,ti OR 'mobile app':ab,ti OR 'mobile phone':ab,ti OR 'mobile phones':ab,ti OR mhealth:ab,ti OR 'm-health':ab,ti OR 'mobile health':ab,ti OR 'mobile electronic device':ab,ti OR 'mobile technolog':ab,ti OR 'mobile communication':ab,ti OR 'mobile computing':ab,ti OR network:ab,ti OR online:ab,ti OR 'online intervention':ab,ti OR 'online interventions':ab,ti OR platform:ab,ti OR 'personal computer':ab,ti OR 'personal digital assistant':ab,ti OR qq:ab,ti OR remote:ab,ti OR smartphone:ab,ti OR 'smart phone':ab,ti OR 'social media':ab,ti OR 'social networking':ab,ti OR telehealth:ab,ti OR 'tele-health':ab,ti OR telephone:ab,ti OR telemedicine:ab,ti OR 'tele-medicine':ab,ti OR 'tele-care':ab,ti OR telecare:ab,ti OR telecommunication:ab,ti OR telemonitor:ab,ti OR 'tele-monitor':ab,ti OR telemonitoring:ab,ti OR twitter:ab,ti OR web:ab,ti OR 'web-based':ab,ti OR website:ab,ti OR wireless:ab,ti OR wechat:ab,ti  #3 'randomized controlled trial'/exp OR 'randomized controlled trials as topic'/exp OR 'controlled clinical trial'/exp OR 'controlled clinical trials as topic'/exp OR 'clinical trials, randomized':ta,ab OR 'trials, randomized clinical':ta,ab OR 'controlled clinical trials, randomized':ta,ab OR rct:ta,ab OR 'clinical trials':ta,ab OR 'controlled clinical trials':ta,ab OR cct:ta,ab  #4 #1 AND #2 AND #3 | 617 |
| the Cochrane library (CENTRAL) | #1 [Diabetes, Gestational] explode all trees OR (Diabetes, Pregnancy-Induced OR Diabetes, Pregnancy Induced OR Diabetes Mellitus, Gestational OR Diabetes, Gestational OR diabetes in pregnancy OR Gestational Diabetes OR Gestational Diabetes Mellitus OR GDM OR maternal diabetes OR Pregnancy-Induced Diabetes OR pregnancy diabetes mellitus):ti,ab,kw  #2 [Mobile Applications] explode all trees OR [Telemedicine] explode all trees OR [Internet] explode all trees OR [Computers] explode all trees OR [Telecommunications] explode all trees OR [Online Systems] explode all trees OR [Software] explode all trees OR [Wireless Technology] explode all trees OR [Cell Phone] explode all trees OR (app OR apps OR application OR applications OR ipad OR blog OR blogging OR computer OR computer interface OR cell phones OR cell phone OR cellular phone OR digital OR digital health OR digital-health OR ehealth OR e-health OR e-mail OR electronic OR E-learning OR Facebook OR health, mobile OR health technolog OR health app OR Internet OR Internet forum OR iphone OR i phone OR i-phone OR ipad OR i pad OR i-pad OR laptop OR linkedin OR mobile OR mobile application OR mobile apps OR mobile app OR mobile phone OR mobile phones OR mhealth OR m-health OR mobile health OR mobile electronic device OR mobile technolog OR mobile communication OR mobile computing OR network OR online OR online intervention OR online interventions OR platform OR personal computer OR personal digital assistant OR QQ OR remote OR smartphone OR smart phone OR social media OR social networking OR telehealth OR tele-health OR telephone OR telemedicine OR tele-medicine OR tele-care OR telecare OR telecommunication OR telemonitor OR tele-monitor OR telemonitoring OR twitter OR web OR web-based OR website OR wireless OR WeChat):ti,ab,kw  #3 [Randomized Controlled Trial] explode all trees OR [Randomized Controlled Trials as Topic] explode all trees OR [Controlled Clinical Trial] explode all trees OR [Controlled Clinical Trials as Topic] explode all trees OR (clinical trials, randomized OR trials, randomized clinical OR controlled clinical trials, randomized OR rct OR clinical trials OR controlled clinical trials OR cct):ti,ab,kw  #4 #1 AND #2 AND #3 | 661 |
| Web of Science | TS = (Diabetes, Pregnancy-Induced OR Diabetes, Pregnancy Induced OR Diabetes Mellitus, Gestational OR Diabetes, Gestational OR diabetes in pregnancy OR Gestational Diabetes OR Gestational Diabetes Mellitus OR GDM OR maternal diabetes OR Pregnancy-Induced Diabetes OR pregnancy diabetes mellitus) AND  TS = (app OR apps OR application OR applications OR ipad OR blog OR blogging OR computer OR computer interface OR cell phones OR cell phone OR cellular phone OR digital OR digital health OR digital-health OR ehealth OR e-health OR e-mail OR electronic OR E-learning OR Facebook OR health, mobile OR health technolog OR health app OR Internet OR Internet forum OR iphone OR i phone OR i-phone OR ipad OR i pad OR i-pad OR laptop OR linkedin OR mobile OR mobile application OR mobile apps OR mobile app OR mobile phone OR mobile phones OR mhealth OR m-health OR mobile health OR mobile electronic device OR mobile technolog OR mobile communication OR mobile computing OR network OR online OR online intervention OR online interventions OR online system OR platform OR personal computer OR personal digital assistant OR QQ OR remote OR smartphone OR smart phone OR social media OR social networking OR Software OR telehealth OR tele-health OR telephone OR telemedicine OR tele-medicine OR tele-care OR telecare OR telecommunication OR telemonitor OR tele-monitor OR telemonitoring OR twitter OR web OR web-based OR website OR wireless OR wireless technology OR WeChat) AND  TS = (Randomized Controlled Trial OR Randomized Controlled Trials as Topic OR Controlled Clinical Trial OR Controlled Clinical Trials as Topic OR clinical trials, randomized OR trials, randomized clinical OR controlled clinical trials, randomized OR rct OR clinical trials OR controlled clinical trials OR cct) | 1326 |
| CINAHL | MH Diabetes, Gestational OR AB (Diabetes, Pregnancy-Induced OR Diabetes, Pregnancy Induced OR Diabetes Mellitus, Gestational OR Diabetes, Gestational OR diabetes in pregnancy OR Gestational Diabetes OR Gestational Diabetes Mellitus OR GDM OR maternal diabetes OR Pregnancy-Induced Diabetes OR pregnancy diabetes mellitus)  AND MH Mobile applications OR MH Telemedicine OR MH internet OR MH Computers OR MH Telecommunications OR MH online system OR MH Software OR MH wireless technology OR MH cell phones OR AB (app OR apps OR application OR applications OR ipad OR blog OR blogging OR computer OR computer interface OR cell phones OR cell phone OR cellular phone OR digital OR digital health OR digital-health OR ehealth OR e-health OR e-mail OR electronic OR E-learning OR Facebook OR health, mobile OR health technolog OR health app OR Internet OR Internet forum OR iphone OR i phone OR i-phone OR ipad OR i pad OR i-pad OR laptop OR linkedin OR mobile OR mobile application OR mobile apps OR mobile app OR mobile phone OR mobile phones OR mhealth OR m-health OR mobile health OR mobile electronic device OR mobile technolog OR mobile communication OR mobile computing OR network OR online OR online intervention OR online interventions OR platform OR personal computer OR personal digital assistant OR QQ OR remote OR smartphone OR smart phone OR social media OR social networking OR telehealth OR tele-health OR telephone OR telemedicine OR tele-medicine OR tele-care OR telecare OR telecommunication OR telemonitor OR tele-monitor OR telemonitoring OR twitter OR web OR web-based OR website OR wireless OR WeChat)  AND MH randomized controlled trials OR MH Randomized Controlled Trials as Topic OR MH Controlled Clinical Trial OR MH Controlled Clinical Trials as Topic OR AB (Clinical Trials, Randomized OR Trials, Randomized Clinical OR Controlled Clinical Trials, Randomized OR RCT OR Clinical Trials OR Controlled Clinical Trials OR CCT) | 33 |
| PsycINFO | AB (Diabetes, Pregnancy-Induced OR Diabetes, Pregnancy Induced OR Diabetes Mellitus, Gestational OR Diabetes, Gestational OR diabetes in pregnancy OR Gestational Diabetes OR Gestational Diabetes Mellitus OR GDM OR maternal diabetes OR Pregnancy-Induced Diabetes OR pregnancy diabetes mellitus) AND  AB (app OR apps OR application OR applications OR ipad OR blog OR blogging OR computer OR computer interface OR cell phones OR cell phone OR cellular phone OR digital OR digital health OR digital-health OR ehealth OR e-health OR e-mail OR electronic OR E-learning OR Facebook OR health, mobile OR health technolog OR health app OR Internet OR Internet forum OR iphone OR i phone OR i-phone OR ipad OR i pad OR i-pad OR laptop OR linkedin OR mobile OR mobile application OR mobile apps OR mobile app OR mobile phone OR mobile phones OR mhealth OR m-health OR mobile health OR mobile electronic device OR mobile technolog OR mobile communication OR mobile computing OR network OR online OR online intervention OR online interventions OR online system OR platform OR personal computer OR personal digital assistant OR QQ OR remote OR smartphone OR smart phone OR social media OR social networking OR Software OR telehealth OR tele-health OR telephone OR telemedicine OR tele-medicine OR tele-care OR telecare OR telecommunication OR telemonitor OR tele-monitor OR telemonitoring OR twitter OR web OR web-based OR website OR wireless OR wireless technology OR WeChat) AND  AB (Randomized Controlled Trial OR Randomized Controlled Trials as Topic OR Controlled Clinical Trial OR Controlled Clinical Trials as Topic OR clinical trials, randomized OR trials, randomized clinical OR controlled clinical trials, randomized OR rct OR clinical trials OR controlled clinical trials OR cct) | 17 |
