# Supplementary material for: Web-Based Interventions for Pregnant Women With Gestational Diabetes Mellitus: Systematic Review and Meta-analysis
Source: J Med Internet Res. 2023 Jan 19;25:e36922. doi: 10.2196/36922 (PMC9896357; doi:10.2196/36922)
Supplement: Multimedia Appendix 3 [file jmir_v25i1e36922_app3.pdf]

Assessment for the methodological quality.

First author, Year	Selection bias	Study design	Confounders	Blinding	Data collection methods	Withdrawals and dropouts	Overall quality [b]
Al-Ofi et al., 2019	Strong	Strong	Strong	Moderate [a]	Strong	Strong	Strong
Borgen et al., 2019	Moderate	Strong	Strong	Moderate	Strong	Strong	Strong
Bromuri et al., 2016	Strong	Strong	Strong	Weak	Strong	Strong	Moderate
Carolan-Olah et al., 2019	Strong	Strong	Strong	Moderate [a]	Strong	Strong	Strong
Carral et al., 2015	Strong	Strong	Weak	Moderate [a]	Strong	Strong	Moderate
Garnweidner-Holme et al., 2020	Moderate	Strong	Strong	Weak	Strong	Strong	Moderate
Ghaderi et al., 2019	Weak	Strong	Strong	Weak	Strong	Strong	Weak
Ghaderi et al., 2022	Weak	Strong	Strong	Weak	Strong	Strong	Weak
Ghasemi et al., 2021	Strong	Strong	Strong	Weak	Strong	Strong	Moderate
Guo et al., 2019	Strong	Strong	Strong	Weak	Strong	Strong	Moderate
Homko et al., 2007	Weak	Strong	Strong	Weak	Strong	Strong	Weak
Homko et al., 2012	Weak	Strong	Strong	Moderate [a]	Strong	Strong	Moderate
Huang et al., 2021	Strong	Strong	Strong	Moderate [a]	Strong	Strong	Strong
Kim et al., 2019	Strong	Strong	Strong	Weak	Strong	Moderate	Moderate
Lemelin et al., 2020	Strong	Strong	Weak	Weak	Strong	Strong	Weak
Mackillop et al., 2018	Strong	Strong	Strong	Weak	Strong	Strong	Moderate
Miremberg et al., 2018	Strong	Strong	Strong	Moderate	Strong	Strong	Strong
Rasekaba et al., 2018	Moderate	Strong	Strong	Moderate	Strong	Strong	Strong
Sayakhot et al., 2016	Strong	Strong	Strong	Weak	Strong	Strong	Moderate
Simsek-Cetinkaya et al., 2022	Strong	Strong	Strong	Weak	Strong	Strong	Moderate
Sung et al., 2019	Strong	Strong	Strong	Weak	Strong	Strong	Moderate
Tian et al., 2021	Strong	Strong	Strong	Weak	Strong	Strong	Moderate
Yang et al., 2018	Strong	Strong	Strong	Moderate [a]	Strong	Strong	Strong
Yew et al., 2021	Strong	Strong	Strong	Weak	Strong	Strong	Moderate
Zhuo et al., 2022	Strong	Strong	Strong	Weak	Strong	Strong	Moderate

[a]: Non-blind trial. The aspect of blinding was rated as "moderate" because only objective outcomes were assessed, which were relatively less likely to be affected by blinding.

[b]: Strong represented that a study received no weak ratings. Moderate represented that a study received one weak rating. Weak represented that a study received two or more weak ratings.
