# Supplementary material for: Web-Based Interventions for Pregnant Women With Gestational Diabetes Mellitus: Systematic Review and Meta-analysis
Source: J Med Internet Res. 2023 Jan 19;25:e36922. doi: 10.2196/36922 (PMC9896357; doi:10.2196/36922)
Supplement: Multimedia Appendix 4 [file jmir_v25i1e36922_app4.pdf]

Characteristics of the 25 studies included in this systematic review.

**[ID] Study, year, country**	**Study design**	**Gestational weeks at allocation**	**Diagnostic criteria of GDM**	**Sample size (IG/CG)**	**Mean age (IG/CG) (Mean ± SD)**	**Intervention group**	Abstract of the intervention characteristic				**Control group**	**Primary outcome**<br>**Secondary outcome**<br>(1) Maternal behavioral outcome;<br>(2) Maternal cognitive and attitudes outcome;<br>(3) Maternal mental health;<br>(4) Maternal and neonatal clinical outcome;<br>(5) Medical service utilisation and cost.	**Adverse event**	**Attrition rate**
							**Duration**	**Main Technology**	**Interactivity (Interactivity personnel)**	**Format**				
[38] Al-Ofi, 2019, Saudi Arabia	RCT	24-28 weeks of gestation	IADPSG (75g OGTT)	IG=27<br>CG=30	IG: 32.5 ±5.80<br>CG: 32.4 ±5.30	1. Name: Tele-GDM.<br>2. Detailed regimen description: A Smartphone-Glucometer and a Glucomail app were installed in participants' phones. Tele-GDM was provided via Glucomail app. Four main contents: (1) Monitor blood glucose and weight gain: participants downloaded their daily blood glucose readings and weight weekly and then reviewed by diabetic care team. The app could alert for hyperglycaemia or hypoglycaemia. If necessary, appropriate dietary advice was delivered to patients by short message; (2) A feedback questionnaire about factors affecting GDM was assessed weekly; (3) Conversation map was used for proactive communication between healthcare professionals and patients; (4) An automated message including some useful facts about body changes, baby size and growth stage, healthy food, and what to avoid during pregnancy was sent to patient weekly according to their due date.	From baseline to 6 weeks post-delivery (around 15-22 weeks)	Mobile app	Interactive intervention (Bidirectional interaction between participants and healthcare professionals)	Non-personalized format	Routine antenatal care: maintaining routine clinic visit and receiving an assessment of dietary structure and appropriate dietary recommendations.	PO: HbA1c (-), FBG (-), 2hBG (+).<br>SO:<br>(1) NR.<br>(2) NR.<br>(3) NR.<br>(4) NR.<br>(5) NR.	NR	5%

[53]<br>[a]Borgen, 2019, Norway	RCT	<33 weeks of gestation	The definition of GDM in the Norwegian guidelines.	IG=115<br>CG=123	Total: >18	1. Name: Pregnant+ smartphone app.<br>2. Detailed regimen description: The Pregnant+ app was download from the Apple Store or Google Play and installed in participants' phones. Women could use the app as needed. Four main icons: (1) Blood glucose: transfering the blood glucose levels and providing a graphical representation; (2) Physical activity: gave written examples, illustrated by images, of how to perform some of activities adapted for pregnant women; (3) Food and beverages: 10 GDM-specific dietary recommendations; (4) Diabetes information: consisted of general information about GDM.	From baseline to about 36 weeks of gestation. (around>3 weeks)	Mobile app	Non-interactive intervention	Non-personalized format	Routine antenatal care: consuling with nurses every 1-2 weeks, receiving information about diet, and recording the blood glucose levels on a paper diary.	PO: NR.<br>SO:<br>(1) NR.<br>(2) NR.<br>(3) NR.<br>(4) ① (-), ② (-), ③ (-), ④ (-), ⑤ (NR), ⑥ (-), ⑦ (-), ⑧ (-), ⑨ (-), ㉙ (-), ㉚ (-).<br>(5) NR.	No adverse events were reported.	5%
[40]<br>[a]Garnweidner-Holme, 2020, Norway												PO: NR.<br>SO:<br>(1) Healthy diet behaviors (measured by a self-designed 9 domains healthy diet questionnaire) (-).<br>(2) NR.<br>(3) NR.<br>(4) NR.<br>(5) NR.	No adverse events were reported.	19%
[19]<br>Bromuri, 201	RCT	24-32 weeks of gestation	Not reported.	IG=12<br>CG=12	IG: 33.0 ± 5.0<br>CG: 32.0	1. Name: Personal health system.<br>2. Detailed regimen description: The types of clients of the Personal health system included website and android app. Patients entered blood glucose levels, medicine taken and any symptoms related to GDM into the system and could visualize	From baseline to delivery. (around	Mobile app or webs	Interactive intervention (Bidirectional interaction between	Non-personalized format	Routine antenatal care: maintaining routine clinic visit and recording the blood glucose	PO: FBG (+), 2hBG (+).<br>SO:<br>(1) Compliance with SMBG-Frequency of SMBG per patient (+).	Hyperglycaemi	0%

6, Switzerland					±4.0	their informations, looked at the entered vales and corrected mistyped values. The system would trigger alerts to medical doctors when detecting hyperglycaemia and hypoglycaemia. The consultations content page allowed tracking the contacts between caregivers and patients.	5-16 weeks)	ite	participants and healthcare professionals)		levels on a paper diary.	(2) NR.<br>(3) NR.<br>(4) ㉙ (-), ㉚ (-).<br>(5) NR.	c episodes (-).	
[54] [b]Carolan-Olah, 2019, Australia	RCT	28-32 weeks of gestation	Not reported.	IG=52<br>CG=58	Total: 31.7 ±6.0	1. Name: Website education.<br>2. Detailed regimen description: Women were given the uniform resource location link and log-in password for the website after the first visit, and they could access the website as often as they wished at home. The four modules: (1) Healthy food choices; (2) Healthy habits/healthy lifestyle; (3) Emotions, family and food; (4) Testing blood glucose levels. The four information resources: (1) What is gestational diabetes? (2) Healthy eating and exercise in GDM; (3) What to do if you're still hungry? (4) A guide to healthy shopping. Each section was followed by a quiz and women could check their understanding and correct their responses. Information was presented in a simple manner with a single message per slide and numerous photos and pictures.	From baseline to 6-8 weeks post-delivery (around 11-20 weeks)	Website	Non-interactive intervention	Non-personalized format	Routine education: a single class lasting 1.5 hours run by diabetes educators. The content included instruction on SMBG, diet, exercise and healthy lifestyle.	PO: NR.<br>SO:<br>(1) NR.<br>(2) NR.<br>(3) NR.<br>(4) ⑧ (-), ⑩ (-).<br>(5) NR.	NR	5%
[25] [b]Sayakhot, 2016, Australia												PO: NR.<br>SO:<br>(1) NR.<br>(2) GDM knowledge (measured by The Diabetes Knowledge Scale): BG test (+), managing when hungry in between meals (+), controlling GDM by changing to a healthy diet and exercise (+).<br>(3) NR.	NR	11%

												(4) NR.<br>(5) NR.		
[57] Carral, 2015, Spain	CCT	<30 weeks of gestation	Not reported.	IG=30<br>CG=47	IG: 34.9 ± 3.9<br>CG: 33.2 ± 4.9	1. Name: Web-based system (DiabeTIC).<br>2. Detailed regimen description: DiabeTIC allowed participants to send blood glucose values, insulin doses administrated, carbohydrate rations consumed and other health data remotely, download the documents of interest from the website library and the treatment reports as well as bidirectional communication between health professionals and patients. Health professionals could view and modify insulin treatment and obtain metabolic control statistics. The system also alerted the professional every 2 weeks for monitoring glycemic control and sending treatment decision to participants by e-mail and short message. Participants attended clinic visit every 6–8 weeks for glucose control evaluation.	From baseline to delivery. (around > 7 weeks)	Web site	Interactive intervention (Bidirectional interaction between participants and healthcare professionals)	Non-personalized format	Routine antenatal care: receiving information about a healthy diet and SMBG, and attending clinic visit every 2-3 weeks for glucose control evaluation.	PO: HbA1c (-).<br>SO:<br>(1) NR.<br>(2) NR.<br>(3) NR.<br>(4) ⑤ (-), ⑪ (-), ⑫ (-), ⑬ (-), ⑭ (-), ⑮ (-), ⑯(-), ⑰ (-), ⑱ (-), ㉙ (+).<br>(5) Frequency of medical service utilisation-The average number of GDM unit visits (+).	NR	11%
[55] [c]Ghaderi, 2019, Iran	CCT	22-32 weeks of gestation	Not reported.	IG=45<br>CG=45	Total: 18-40	1. Name: GDM app education.<br>2. Detailed regimen description: The GDM app was installed on the smartphones and the usage instructions were taught. There were eight encrypted files in the app and the passwords were provided twice a week. The educational contents included the GDM definition, risk factors, consequences, diagnosing, blood glucose measurement methods, nutrition, physical activity, medicine-based treatments, weaning, stress management, T2DM screening after childbirth, the definition and prevention of T2DM, and statistics about getting T2DM in women with GDM.	Around 4 weeks	Mobile app	Non-interactive intervention	Non-personalized format	Routine antenatal care: using educational booklets as well as official and non-organised education.	PO: NR.<br>SO:<br>(1) NR.<br>(2) Risk-perception of type 2 diabetes (measured by a risk perception survey for developing-diabetes) (+).<br>(3) NR.<br>(4) NR.<br>(5) NR.	NR	3%
[43] [c]Ghaderi,												PO: NR.<br>SO:<br>(1) NR.<br>(2) Self-efficacy (measured by the	NR	3%

2022, Iran												Self-efficacy scale for diabetic patients) (+);<br>(3) NR.<br>(4) NR.<br>(5) NR.		
[41] Ghasemi, 2021, Iran	RCT	24-26 weeks of gestation	Not reported.	IG=42<br>CG=42	IG: 30.52±2.70<br>CG: 30.88±1.54	1. Name: WhatsApp social network.<br>2. Detailed regimen description: Participants received a booklet containing the information needed by diabetic mothers, contacted with the counselor on private page in WhatsApp, and then received counseling of using GATHER (G = Greeting, A = Ask, T = Tell, H = Help, R = Return) method once a week in consecutive four 45-min sessions. The sessions discussed on 5 categories of GDM self-care: diet, physical activity, medications, blood glucose control and fetal health. The counselor was always available online to answer the questions, address concerns, and provide personal advice via the private page.	From baseline to about 36 weeks of gestation. (around 10-12 weeks)	Mobile app	Interactive intervention (Bidirectional interaction between participants and midwifes)	Non-personalized format	Routine antenatal care: no details.	PO: NR.<br>SO:<br>(1) Overall self-care behaviors (measured by a revised self-care behaviors questionnaire) (+).<br>(2) NR.<br>(3) NR.<br>(4) ② (-), ⑤ (-), ⑬ (-).<br>(5) NR.	NR	0%
[26] Guo, 2019, China	RCT	24-28 weeks of gestation	IADPSG (75g OGTT)	IG=64<br>CG=60	IG: 31.2±4.1<br>CG: 30.6±3.1	1. Name: Dnurse app.<br>2. Detailed regimen description: Dnurse app was installed in participants' smartphones. It could be used for uploading blood glucose data, which was subsequently viewed by doctors. When an abnormal blood glucose value was uploaded, participants were notified and the underlying cause was analyzed. Participants could learn more about GDM by reading information about diet, physical activity, medicine, and diabetes education in Dnurse app. The education nurse provided two hours online individualized instruction every night and answered any questions about GDM.	From baseline to delivery. (around 9-16 weeks)	Mobile app	Interactive intervention (Bidirectional interaction between participants and education nurse)	Personalized format: provided personalized instruction	Routine antenatal care: receiving information about diet and physical activity and recording the blood glucose levels on a paper diary. The physician adjusted the treatment plan as needed. One	PO: HbA1c (+), FBG (+), 2hBG (+).<br>SO:<br>(1) Compliance with SMBG-Compliance (%) (+).<br>(2) NR.<br>(3) NR.<br>(4) ② (-), ③ (-), ④ (NR), ⑤ (-), ⑧ (-), ⑪ (+), ⑫ (-), ⑱ (-), ⑲ (-).<br>(5) Frequency of medical service utilisation-The average number of	NR	0%

											clinic visit per week for three consecutive weeks for the initial visits and once every 2–4 weeks after the blood glucose had stabilized.	outpatient service (+).		
[59] Homko, 2007, USA	RCT	<33 weeks of gestation	Carpenter and Coustan	IG=34<br>CG=29	IG: 29.8 ± 6.6<br>CG: 29.2 ± 6.7	1. Name: ITSMyHealthrecord®<br>2. Detailed regimen description: ITSMyHealthrecord® composed of a secure Internet server and a database. It allowed women to input blood glucose values, foetal movement counts, insulin doses and any episodes of hypoglycaemia, send the data at least 3 times per week, and received personalized feedback from health care providers. The patients could review all entered data on the data entry page. The web interface also had links to educational resources about GDM. The messaging between clinicians and patients was also allowed.	From baseline to delivery. (around > 4 weeks)	Web site	Interactive intervention (Bidirectional interaction between participants and healthcare professionals)	Personalized format: received personalized feedback	Routine antenatal care: receiving clinical evaluation, individualized diet counseling and diabetes education, and recording blood glucose levels, fetal movement counting, insulin doses and episodes of hypoglycemia on a paper diary. Women attended the clinic visit biweekly up to 35 weeks of gestation, followed by weekly visits until delivery.	PO: HbA1c (-), FBG (-), 2hBG (-).<br>SO:<br>(1) NR.<br>(2) Self-efficacy (measured by the Diabetes Empowerment Scale): managing the psychosocial aspects of diabetes (+), assessing dissatisfaction and readiness to change (+).<br>(3) NR.<br>(4) ⑤ (-), ⑨ (-), ⑫ (-), ⑬ (-), ⑭ (-), ⑮ (-), ⑯ (-), ⑱ (-), ⑳ (-), ㉑ (-), ㉒ (-), ㉓ (-), ㉔ (-), ㉕ (-), ㉙ (+), ㉚ (-).<br>(5) NR.	NR	10%

[58] Homko, 2012, USA	RCT	<33 weeks of gestation	Carpenter and Coustan	IG=40 CG=40	IG: 30.0 ± 7.5 CG: 30.3 ± 6.0	1. Name: Enhancement ITSMyHealthrecord® 2. Detailed regimen description: Enhancement ITSMyHealthrecord® composed of a secure Internet server, an interactive voice response-enabled phone system, and a database. The interactive voice response addressed the technical difficulties with hardware installation and maintenance. The functions of the Enhancement ITSMyHealthrecord® were similar as the ITSMyHealthrecord®. Additionally, women were also provided feedback, emotional support, and reinforcement regarding diabetes self-management (such as diet and activity) with each transmission and received a brief educational message each time they accessed the system by phone or Internet. This system could automat reminders for patients to transmit data.	From baseline to delivery. (around > 4 weeks)	Web site	Interactive intervention (Bidirectional interaction between participants and healthcare professionals)	Personalized format: received personalized feedback	Routine antenatal care: receiving clinical evaluation, individualized diet counseling and diabetes education, and recording blood glucose levels, fetal movement counting, insulin doses and episodes of hypoglycemia on a paper diary. Women attended the clinic visit biweekly up to 35 weeks of gestation, followed by weekly visits until delivery.	PO: FBG (-), 2hBG (-). SO: (1) NR. (2) NR. (3) NR. (4) ⑤ (-), ⑨ (-), ⑫ (-), ⑬ (-), ⑭ (-), ⑮ (-), ⑯ (-), ⑱ (-), ⑳ (-), ㉑ (-), ㉒ (-), ㉓ (-), ㉔ (-), ㉙ (-), ㉚ (-). (5) NR.	NR	7%

[42] [d]Huang, 2021, China	RCT	23-31 weeks of gestation	IADPSG (75g OGTT)	IG=147 CG=162	IG: 31.23±4.21 CG: 30.93±4.48	1. Name: WeChat Group Management.<br>2. Detailed regimen description: Researchers issued a task card in the WeChat group to pinpoint the basic requirements (including diet advice, examples of meals and physical activity rules) every Monday. Patients performed self-management according to the basic criteria and shared photos of their meals and snacks, daily physical activity and experience regarding blood glucose control. Then researchers gave individualized guidance for self-management. On weekends, the researchers prepared lessons and articles (including rudimentary knowledge, disease management, psychology and past cases) for group members. Sharing of learning experiences and notes in the form of peer interactions and support groups were encouraged. The answers of any questions regarding the project, pregnancy or GDM could be sought from the group chat.	From baseline to delivery. (around 6-17 weeks)	Mobile app	Interactive intervention (Bidirectional interaction between participants and a clinical team, as well as **peer groups interaction**)	Personalized format: provided personalized guidance for self-management	Routine antenatal care: receiving information about self-management of GDM and maintaining routine clinic visit once every 2 weeks.	PO: NR.<br>SO:<br>(1) Compliance with SMBG-Compliance (%) (-).<br>(2) NR.<br>(3) NR.<br>(4) NR.<br>(5) NR.	NR	13%
[30] [d]Tian, 2021, China												PO: NR.<br>SO:<br>(1) NR.<br>(2) NR.<br>(3) NR.<br>(4) ④ (-), ⑤ (-), ⑧ (-), ⑩ (-), ⑬ (-), ⑳ (-).<br>(5) NR.	NR	13%

[27] Kim, 2019, Korea	CCT	24-28 weeks of gestation	Not reported.	IG=22 CG=22	IG: 35.1 ± 3.8 CG: 36.4 ± 3.1	1. Name: DIETEX website.<br>2. Detailed regimen description: The participants registered in DIETEX website. A self-care program was provided via DIETEX website. Participants used the Passometer application to measure the number of steps daily, and recorded FBG, steps and weight in DIETEX website more than once a week. The researcher provided customized education weekly in DIETEX website. The education contents: (1) blood glucose management; (2) Meal therapy; (3) Physical activity; (4) Weight management.	From baseline to delivery. (a little more than 12 weeks on average)	Web site	Non-interactive intervention	Personalized format: provided personalized education on diet and exercise	Routine education: one session of nutrition education	PO: HbA1c (+), FBG (-), 1hBG (-).<br>SO:<br>(1) Overall self-care behaviors (measured by a 15 questions tool) (-).<br>(2) NR.<br>(3) Depression (measured by the Self-rating Depression Scale) (-), Anxiety (measured by the State-Trait Anxiety Inventory) (+).<br>(4) NR.<br>(5) NR.	NR	25%
[15] Lemelin, 2020, Canada	CCT	21-31 weeks of gestation	IADPSG (75g OGTT)	IG=80 CG=81	IG: 32.9 ± 4.3 CG: 32.5 ± 4.6	1. Name: THCa system.<br>2. Detailed regimen description: Participants accessed to the website patient portal via a computer/tablet/phone. In the THCa system, they could recorded their health status and blood glucose values, accessed a health library to review specific teachings around GDM management, viewed a weekly summary of blood glucose values, and interactively contacted the health team through the messaging system of the platform. If the blood glucose values out of the normal range, potential reasons analyzing and coaching were delivered directly through the system by the clinical team or automatically from a set of pre-programmed algorithms activated. Then, clinicians would receive alerts and adjust therapy. At the same tine, registered nurses assessed the glycemic control on weekdays, reviewed all data and charts with the medical team at least every 2 weeks, and transmited medical conclusions or modifications to	From baseline to delivery. (around 6-19 weeks)	Web site	Interactive intervention (Bidirectional interaction between participants and a health care team including doctors and registered nurses)	Non-personalized format	Routine antenatal care: the frequency of clinic visit was based on clinical judgment without predetermined number.	PO: NR.<br>SO:<br>(1) NR.<br>(2) Satisfaction with care-satisfaction with educational support (measured by the10-Likert scale) (+).<br>(3) NR.<br>(4) ③ (-), ⑤ (-), ⑥ (-), ⑦ (-), ⑧ (-), ⑩ (-), ⑫ (-), ⑬ (-), ⑭ (-), ⑮ (-), ⑯ (-), ⑰ (-), ⑱ (-), ⑲ (-), ⑳ (-), ㉔ (-), ㉖ (-).<br>(5) Frequency of medical service utilisation-The average number of medical visits (+); Medical service	NR	0%

						participants.						costs-Total average costs for GDM management (+).		
[16] Mackillop, 2018, United Kingdom	RCT	<35 weeks of gestation	IADPSG (75g OGTT)	IG=103<br>CG=103	IG: 33.9 ± 5.5<br>CG: 33.0 ±5.6	1. Name: GDm-health management system.<br>2. Detailed regimen description: Participants recorded, taged, and reviewed blood glucose readings via GDm-health app. Then, a diabetes midwife reviewed the blood glucose readings at least three times a week. The system generated an alert if a participant was not recording a predefined number of blood glucose readings per week or more glucose testing strips were needed. A short message containing advice about diet, dose adjustments of hypoglycemic medications and encouragement message was sent to the participant between clinic visits via the website. Participants attended the clinic visit every 4-8 weeks.	From baseline to delivery. (around>2 weeks)	Mobile app	Non-interactive intervention	Non-personalized format	Routine antenatal care: recording the blood glucose values in a paper diary and attending the clinic visit every 2-4 weeks.	PO: HbA1c (-), FBG (-), 2hBG (-).<br>SO:<br>(1) Compliance with SMBG-Frequency of SMBG per day (+).<br>(2) Satisfaction with care-overall satisfaction with care (measured by the Oxford Maternity Diabetes Treatment Satisfaction Questionnaire) (+).<br>(3) NR.<br>(4) ② (-), ③ (NR), ④ (NR), ⑤ (NR), ⑥ (-), ⑦ (NR), ⑨ (-), ⑪ (-), ⑫ (-), ⑬ (-), ⑭ (-), ⑮ (-), ⑯ (-), ⑱ (-), ⑲ (-), ㉓ (-).<br>(5) Frequency of medical service utilisation-The average number of hospital doctor visits (-); Medical service costs-The average costs antenatal care (-).	NR	1 %
[56] Miremberg 201	RCT	<34 weeks of gestation	Carpenter and Coustan. Additionally, women with one abnormal	IG=60<br>CG=60	IG: 31.7 ±4.2<br>CG: 32.0 ±6.3	1. Name: Glucosebuddy app.<br>2. Detailed regimen description: Glucosebuddy app was installed in participants' smartphones. Participants documented their blood glucose values in the app, which generated a daily report transmitted by e-mail every evening to the research database. Then, participants received a individualized feedback	From baseline to the last prenatal visit.	Mobile app	Interactive intervention (Bidirectional interaction between participants	Personalized format: provided personalized	Routine antenatal care: receving the information about the proper use of the glucometer, dietary, physical	PO: FBG (+), 1hBG (+).<br>SO:<br>(1) Compliance with SMBG-Compliance (%) (+).<br>(2) NR.<br>(3) NR.	NR	5 %

8, Israel			value in the OGTT and an additional risk factor (obesity, GDM in a previous pregnancy, or a first-degree family member with diabetes mellitus type 2) were also diagnosed with GDM.			every evening, which included reassurance and positive messaging, dietary tips in attempts to optimize specific off-target measurements, modifications in insulin treatment, or alerts to reschedule an earlier appointment to the clinic. Participants could use the platform to ask questions regarding any aspect of GDM management and receive immediate answers.	(around>2 weeks)		and a clinic team)	feedback	activity, blood pressure monitoring et al., recording the blood glucose values in a paper diary and attending the clinic visit biweekly up to 35 weeks of gestation, followed by weekly visits until delivery.	(4) ① (-), ② (-), ③ (-), ④ (NR), ⑤ (-), ⑥ (NR), ⑦ (-), ⑨ (-), ⑫ (-), ⑭ (-), ⑮ (-), ⑯ (-), ⑱ (-), ⑲ (-), ㉔ (-), ㉕ (-), ㉖ (-), ㉗ (-), ㉘ (-), ㉙ (+).<br>(5) NR.		
[31] Rasekaba, 2018, Australia	RCT	<35 weeks of gestation	IADPSG (75g OGTT)	IG=61<br>CG=34	IG: 32.5 ±5.0<br>CG: 32.5 ±5.0	1. Name: Online Health Portfolio.<br>2. Detailed regimen description: Participants entered their blood glucose values, insulin dosing, dietary information and symptoms on the Online Health Portfolio. Then, registered nurses reviewed the data at their convenience and provided feedback on individualised GDM care via the inbuilt messaging feature. The inbuilt messaging platform also enabled 2-way messaging between clinicians and patients.	From baseline to delivery. (10 weeks on average)	Website	Interactive intervention (Bidirectional interaction between participants and diabetes education-registered nurses)	Personalized format: provided personalized feedback on GDM care	Routine antenatal care: recording the blood glucose values in a paper diary and attending the clinic visit every 1-2 weeks.	PO: FBG (-), 2hBG (-).<br>SO:<br>(1) Compliance with SMBG-Frequency of SMBG per patient (-).<br>(2) NR.<br>(3) NR.<br>(4) ⑤ (-), ⑥ (NR), ⑦ (-), ⑧ (-), ⑨ (-), ⑫ (-), ⑮ (-).<br>(5) Frequency of medical service utilisation-The average number of medical appointments (-); Medical service costs-The average costs for	No adverse events were reported.	0 %

												medical appointments (-).		
[44] Simsek-Cetinkaya, 2022, Turkey	RCT	GDM between 24-28 weeks of gestation without pregnancy complication or neurological or psychological disease	Not reported.	IG=23 CG=22	Total: 18-45	1. Name: Smartphone-based counseling system<br>2. Detailed regimen description: An app was installed in participants' phones and the usage instructions were taught. The app consisted of 5 sections: (1) A digital education booklet about GDM; (2) Diet compliance tracking: including weight gain, number of meals, SMBG, status of initiation of insulin therapy, and insulin doses; (3) Physical activity monitoring; (4) A platform allowed to ask questions and receive immediate answers regarding any aspect of GDM management. Participants received individualized feedback from the nurse and could communicated with other participants; and (5) An admin page: generating and transmitting a daily report about the patient's documents, sending SMS to remind participants about the lack of data transmission, and sending a warning message to the participants who had high BG levels for 3 days and did not do physical activity 3 days a week or had problems following their diet.	14 weeks	Mobile app	Interactive intervention (Bidirectional interaction between participants and nurses, as well as peer groups interaction)	Personalized format: provided personalized feedback	Routine antenatal care: receving the information about the proper use of the glucometer and insulin, dietary, blood pressure monitoring et al., recording the blood glucose values 4 times a day and attending the clinic visit biweekly up to 34 weeks of gestation, followed by weekly visits until delivery.	PO: FBG (-), 1hBG (-), 2hBG (-).<br>SO:<br>(1) Healthy diet behaviors (measured by a self-compiled physical activity diary) (+), Physical activity (measured by a self-compiled physical activity diary) (+).<br>(2) GDM knowledge (measured by a self-designed 16-item questionnaire) (+).<br>(3) NR.<br>(4) ㉙ (-).<br>(5) NR.	NR	10%
[39] Sung, 2019, Korea	RCT	24-28 weeks of gestation	Carpenter and Coustan.	IG=11 CG=10	IG: 35.0 ±2.76 CG: 31.7 ±4.92	1. Name: Mobile app (Huraypositive Inc).<br>2. Detailed regimen description: Participants received a glucometer with Bluetooth connectivity and an accelerometer to detect physical activity level. A mobile app (Huraypositive Inc) was installed in participants' phones. The app consisted of 4 sections: (1) Clinical data; (2) Nutrition and diet; (3) Medication; (4) Messaging system and information. Participants could used the app to record the blood glucose values and diet,	From baseline to delivery. (around 9-16 weeks)	Mobile app	Interactive intervention (Bidirectional interaction between participants and a health care team	Personalized format: provided personalized medical and	Routine clinical care: biweekly clinic visits up to 36 weeks of gestation, followed by weekly visits until delivery.	PO: NR.<br>SO:<br>(1) Physical activity (measured by a accelerometer) (-).<br>(2) NR.<br>(3) NR.<br>(4) ⑤ (-), ⑪ (-), ⑫ (-), ⑮ (-), ⑯ (-), ⑰ (-).	NR	10%

						receive messages about adequate diet and physical activity weekly, and communicate with health care providers. Health care providers scanned and analyzed the transmitted records and sent return messages with tailored medical and nutritional guidance to participants biweekly.			including an endocrinologist, nurses, and nutritionists)	nutritional guidance		(5) NR.		
[28] Yang, 2018, China	CCT	24-28 weeks of gestation	IADPSG (75g OGTT)	IG=57<br>CG=50	IG: 31.61 ± 4.16<br>CG: 32.22 ± 4.69	1. Name: Wechat GDM-care management system.<br>2. Detailed regimen description: Participants registered in WeChat and entered the WeChat platform management system. Participants had access to the front-end (a WeChat platform) and the doctors had access to the back-end of this system. Participants reported blood glucose values, weight, and ketone via WeChat platform, received information about GDM and individualized dietary and physical activity advice from an obstetrician and qualified dietitian.	From baseline to delivery. (around 9-16 weeks)	Mobile app	Non-interactive intervention	Personalized format: provided personalized dietary advice	Routine clinical care: no details.	PO: FBG (+), 1hBG (-), 2hBG (+).<br>SO:<br>(1) NR.<br>(2) NR.<br>(3) NR.<br>(4) ③ (-), ④ (-), ⑤ (+), ⑧ (-), ⑨ (-), ⑬ (+), ⑭ (-), ⑮ (-), ⑱ (-), ⑳ (-),㉓ (-).<br>(5) NR.	NR	0%
[29] Yew,2021,Singapore	RCT	12-30 weeks of gestation	IADPSG (75g OGTT)	IG=170<br>CG=170	IG: 31.7 ±4.0<br>CG: 32.2 ±4.4	1. Name: Habits-GDM app.<br>2. Detailed regimen description: The Habits-GDM app with passcode protected was installed in participants' smartphones. Smartphone-based lifestyle coaching program in GDM (SMART-GDM) was provided via Habits-GDM app. The program comprises 12 interactive lessons. Participants could go through the lessons at their own pace. Four main parts of the app: (1) Diet: A database of common foods in Singapore was incorporated into Habits-GDM. Participants recorded their diet in the preceding 2-4 hours; (2) SMBG: values were automatically captured into Habits-GDM that interfaces with a glucometer. SMBG reports were generated weekly to monitor progress and were reviewed by the health care teams; (3) Physical activity; (4) Weight tracking: used a Bluetooth weighing scale. The frequency of prompts to weigh themselves	From baseline to 35–37 weeks of gestation. (around 5-25 weeks)	Mobile app	Interactive intervention (Bidirectional interaction between participants and healthcare professionals)	Non-personalized format	Routine antenatal care: receving a single session education lasting 1-1.5 hours run by a diabetes nurse educator and dietitian, and conducting SMBG on a paper diary.	PO: NR.<br>SO:<br>(1) Compliance with SMBG-Frequency of SMBG per week (-).<br>(2) NR.<br>(3) Depression (measured by the Edinburgh Postnatal Depression Scale) (-), Anxiety (measured by the State-Trait Anxiety Inventory) (-).<br>(4) ② (-), ③ (-), ④ (NR), ⑤ (-), ⑥ (-), ⑦ (-), ⑧ (-), ⑨ (-), ⑪ (-), ⑬ (-), ⑭ (-), ⑮ (-), ⑱ (-), ㉓ (-), ㉔ (-), ㉕ (+), ㉗ (-), ㉙ (-).	NR	3%

						increased once Gestational weight gain exceeded the optimal range. There was a messaging platform for communicating with health care professionals and the health care team would respond within 24 hours.						(5) NR.		
[45] Zhuo, 2022, China	RCT	Single pregnancy, GDM between 24-35 weeks of gestation commenced insulin therapy	IADPSG (75g OGTT)	IG=62<br>CG=62	IG: 32.5 (28.0–35.0)<br>CG: 33.0 (28.5–36.5)	1. Name: cCinical pharmacist-led “continuing medical care” (CMC) app<br>2. Detailed regimen description: The CMC app was download and installed in participants' phones. Three main domains: (1) Personal information recording: including their SMBG values, insulin doses, diet and physical exercise; (2) GDM education: diabetes clinical pharmacists are authorized to post educational articles and videos mainly concerning insulin storage information, insulin injection technique, treatment for injection phobia, and measures to cope with insulin injection omissions on to CMC; and (3) A interactive platform: allowing the communication between patients and pharmacists, covering insulin dosage adjustments, recommendations on the management of hyperglycaemia and hypoglycaemia events, medication adherence evaluation, and insulin injection omissions assessment.	Around 9 weeks	Mobile app	Interactive intervention (Bidirectional interaction between participants and pharmacists)	Non-personalized format	Routine antenatal care: recording the blood glucose values in a paper diary and attending the clinic visit every 2 weeks.	PO: NR.<br>SO:<br>(1) Compliance with SMBG-The proportion of participants completed the SMBG task (+);<br>(2) NR.<br>(3) NR.<br>(4) Maternal and neonatal clinical outcome: ⑬ (-), ⑤(+), ⑳ (-), ㉘(-), ㉗ (-), ⑨(+), ⑮ (-), ⑧ (-), ⑱ (-), ㉓ (-), ㉔ (-).<br>(5) NR.	No severe hypoglycemia occurred.	4%

1hBG: 1-hour postprandial blood glucose; 2hBG: 2-hour postprandial blood glucose; CCT: controlled clinical trials; CG: control group; FBG: fasting blood glucose; GDM: gestational diabetes mellitus; HbA1c: glycated haemoglobin; IADPSG: International Association Diabetic Pregnancy Study Group; IG: intervention group; NR: not reported; OGTT: Oral Glucose Tolerance Test; PO: primary outcome; RCT: randomized controlled trials; SMBG: self-monitoring of blood glucose; SO: secondary outcome; T2DM: type 2 diabetes mellitus; (+): Significant between-group difference; (-): Non-significant between-group difference; ①Induction of labor; ②Normal vaginal delivery; ③Assisted vaginal delivery; ④Vaginally delivery; ⑤Caesarean delivery; ⑥ Planned caesarean; ⑦Emergency caesarean; ⑧Macrosomia (≥4000 g); ⑨Admission to neonatal intensive care unit; ⑩Low birth weight (<2500 g); ⑪ Gestational weight gain; ⑫ Gestational weeks at delivery; ⑬ Premature delivery; ⑭ Pre-eclampsia/gestational hypertension; ⑮ Birth weight; ⑯ Large for gestational age; ⑰ Small for gestational age; ⑱ Neonatal hypoglycemia; ⑲ Shoulder dystocia; ⑳ Premature rupture of the membranes; ㉑ 1 minute apgar scores; ㉒ 5 minute apgar scores; ㉓ Neonatal jaundice/hyperbilirubinemia; ㉔ Respiratory morbidity; ㉕ Composite neonatal complication; ㉖ Phototherapy; ㉗ Neonatal death; ㉘ Polyhydramnios; ㉙ Insulin treatment rate; ㉚ Oral antidiabetic drug treatment rate.

[a,b,c,d]: The two corresponding publications were from the same RCT, so the study design, participants characteristics, diagnostic criteria of GDM, sample size, mean age of participants, intervention regimen, and control regimen of two corresponding publications were the same.
